# The degree of enhancer or promoter activity is reflected by the levels and directionality of eRNA transcription

**DOI:** 10.1101/gad.308619.117

**Published:** 2018-01-01

**Authors:** Olga Mikhaylichenko, Vladyslav Bondarenko, Dermot Harnett, Ignacio E. Schor, Matilda Males, Rebecca R. Viales, Eileen E.M. Furlong

**Affiliations:** Genome Biology Unit, European Molecular Biology Laboratory (EMBL), D-69117 Heidelberg, Germany

**Keywords:** developmental enhancers, promoters, eRNA, spatio–temporal expression, ncRNA, embryonic development

## Abstract

Gene expression is regulated by promoters, which initiate transcription, and enhancers, which control their temporal and spatial activity. However, the discovery that mammalian enhancers also initiate transcription questions the inherent differences between enhancers and promoters. Here, we investigate the transcriptional properties of enhancers during *Drosophila* embryogenesis using characterized developmental enhancers. We show that while the timing of enhancer transcription is generally correlated with enhancer activity, the levels and directionality of transcription are highly varied among active enhancers. To assess how this impacts function, we developed a dual transgenic assay to simultaneously measure enhancer and promoter activities from a single element in the same embryo. Extensive transgenic analysis revealed a relationship between the direction of endogenous transcription and the ability to function as an enhancer or promoter in vivo, although enhancer RNA (eRNA) production and activity are not always strictly coupled. Some enhancers (mainly bidirectional) can act as weak promoters, producing overlapping spatio–temporal expression. Conversely, bidirectional promoters often act as strong enhancers, while unidirectional promoters generally cannot. The balance between enhancer and promoter activity is generally reflected in the levels and directionality of eRNA transcription and is likely an inherent sequence property of the elements themselves.

## Abstract (precis)

Here, Mikhaylichenko et al. investigate the transcriptional properties of enhancers during *Drosophila* embryogenesis using characterized developmental enhancers. The authors demonstrate that while the timing of enhancer transcription is correlated with enhancer activity, the levels and directionality of transcription are highly varied among active enhancers and conclude that this is likely an inherent sequence property of the elements themselves.

Transcription in higher eukaryotes is regulated by the interplay of regulatory information encoded in promoters (where gene transcription initiates) and more distal enhancers. Despite sharing some features such as transcription factor (TF)-binding sites, promoters and enhancers have historically been considered as two distinct classes of regulatory elements. The term “promoter” implies the ability to recruit RNA polymerase II (Pol II) and initiate gene expression either at focused transcriptional start sites (TSSs; often called narrow promoters) or through more dispersed regions (broad promoters) (Lenhard et al. 2012; Roy and Singer 2015; Schor et al. 2017; Vo Ngoc et al. 2017). In contrast, enhancers are defined by their ability to activate transcription remotely using a promoter at another distal site and to function in an orientation-independent manner. Naively, enhancers therefore were not expected to have the necessary sequences for transcription initiation themselves. However, transcription has been observed at well-characterized enhancers (Tuan et al. 1992) and more globally (Tuan et al. 1992; De Santa et al. 2010; Kim et al. 2010; Lam et al. 2013). Moreover, Pol II and general TFs can bind to enhancers (Koch et al. 2011), suggesting that they have some inherent promoter activity.

Enhancer transcription and its product, enhancer RNA (eRNA), have been extensively studied in mouse and human cells, where several common properties emerged. Mammalian eRNAs are generally nonpolyadenlyated, low in abundance, unspliced, and retained within the nucleus (De Santa et al. 2010; Kim et al. 2010; Core et al. 2012; Andersson et al. 2014a,b). They are often bidirectionally transcribed (Kim et al. 2010; Melgar et al. 2011; Hah et al. 2013; Kaikkonen et al. 2013; Andersson et al. 2014a), although some unidirectional transcription has also been reported (Ho et al. 2006; Kim et al. 2007; De Santa et al. 2010; Koch et al. 2011). In *Caenorhabditis elegans*, putative enhancers (intergenic TF-bound regions) have bidirectional eRNA transcription but form longer transcripts in the direction of the closest downstream gene (Chen et al. 2013). This resembles some intragenic mammalian enhancers, which also transcribe long noncoding RNA (lncRNA) transcripts in the direction of the gene's transcription (Kowalczyk et al. 2012). This suggests that these long unidirectional eRNAs are a different class from short bidirectional unstable eRNAs, or perhaps there is a continuum of eRNA with different lengths and directionality in between. The ability to transcribe eRNA appears autonomous to the enhancer sequence itself, as eRNA production occurs independently of the transcription of the target gene, at least in the case of the α-globin promoter (Vernimmen 2014). In keeping with this, many transcribed enhancers have Initiator (INR)-like motifs that are positioned at the point of eRNA transcription in both vertebrates (Andersson et al. 2014a) and *C. elegans* (Chen et al. 2013).

The functional role of eRNAs remains unclear (for reviews, see Natoli and Andrau 2012; Lam et al. 2014). Whether it is the process of enhancer transcription that is important (Wilson et al. 1996; Cho et al. 1998; Kaikkonen et al. 2013) or the eRNA itself (Lam et al. 2013; Melo et al. 2013; Li et al. 2014) may vary from one locus or context to another. Alternatively, it may be a nonfunctional consequence of the random engagement of Pol II to open chromatin (Struhl 2007). Nevertheless, there is clear association between enhancer transcription and enhancer activity, as observed at well-characterized enhancers (e.g., in the α-globin [Vernimmen et al. 2007] and β-globin [Tuan et al. 1992; Plant et al. 2001] loci) and globally at putative enhancers following TF stimulation (as shown for p53 [Melo et al. 2013], FoxA1 [Wang et al. 2011], and estrogen receptor [Hah et al. 2013]). Moreover, when stimulating gene expression, transcription of noncoding elements (putative enhancers) often precedes that of the stimulus-induced genes’ expression, as observed in mouse primary neurons (Kim et al. 2010) and macrophages (Kaikkonen et al. 2013). Consistent with this, the timing of Pol II enhancer occupancy is highly predictive of the timing of enhancer activity (Bonn et al. 2012a). This cumulative evidence indicates a strong link between eRNA production, enhancer activity, and gene expression.

This growing evidence indicates that enhancers have some inherent promoter capacity, raising the question of whether promoters reciprocally possess enhancer activity. A number of studies have measured the ability of different genomic elements to function as either enhancers or promoters using massively parallel cell culture-based reporter assays. Measuring promoter activity of thousands of small DNA elements (Nguyen et al. 2016) or across the entire human genome (van Arensbergen et al. 2017) suggests that many enhancers (as well as repetitive elements) can act as weak autonomous promoters, although at levels ∼10-fold lower than that of annotated gene promoters. Conversely, testing promoters for enhancer activity revealed that ∼3% of human elements (Dao et al. 2017) and 4.5% of *Drosophila* elements (Arnold et al. 2013) spanning promoters have in vitro enhancer activity. Interestingly, the percentage increased to 58.4% when testing elements overlapping housekeeping promoters (Arnold et al. 2013; Zabidi et al. 2015), indicating that these regions have different inherent properties. Moreover, the deletion of a number of promoter regions in mammals altered the transcription of distal genes in *cis*, in keeping with an enhancer-like function (Dao et al. 2017; Diao et al. 2017). While indirect effects of the deletions on chromatin topology or other mechanisms cannot be excluded in these studies, the accumulating evidence suggests that some promoters have enhancer activity. Enhancer and promoter activities thereby seem to be properties that can co-occur in the same *cis*-regulatory element. However, measurements of the ability of an element to be activated by an enhancer, termed “enhancer responsiveness,” indicate that promoter and enhancer activities are uncoupled in both their function and underlying sequence motifs (Arnold et al. 2017).

Although much of our understanding of how developmental enhancers function has come from model organisms, including *Drosophila*, enhancer transcription remains largely unexplored in this long-standing model organism. Here, we exploited thousands of characterized developmental enhancers (Gallo et al. 2011; Bonn et al. 2012a; Kvon et al. 2014) and putative enhancers defined by TF co-occupancy (Zinzen et al. 2009; Junion et al. 2012) available in *Drosophila* to characterize eRNA properties during embryogenesis using deeply sequenced PRO-cap (a precision nuclear run-on sequencing variant) and CAGE (cap analysis of gene expression) data. Our results reveal that active developmental enhancers possess a range of eRNA levels and directionality. We explored the relationship between these properties and enhancer and promoter function using a new in vivo assay, simultaneously assessing both activities for the same element within the same embryo. This revealed that the ability of enhancers to function as promoters in vivo is highly correlated to both their level and directionality of eRNA transcription. Intergenic enhancers transcribed predominantly bidirectionally can generally function as weak promoters in both orientations. However, this was not the case for the majority of elements (both enhancers and promoters) with unidirectional transcription: The direction of endogenous transcription was generally correlated to the orientation in which the element can function as a promoter in vivo. Testing gene promoters for enhancer activity revealed that when bidirectionally transcribed, promoters can function as enhancers in vivo, while unidirectional promoters generally cannot. These proximal bidirectional elements appear to function as both an enhancer and a promoter for the same gene, thereby regulating both their spatial pattern and levels of expression. Taken together, our results indicate that there is a continuum of *cis*-regulatory activity, with some elements acting strictly as either an enhancer or a promoter, while others function predominantly as an enhancer with weak promoter activity, and yet others (for example, alternative promoters) have both strong promoter and enhancer activity. This spectrum of activities is highly correlated with the directionality of their eRNA transcription, which likely reflects the underlying sequence properties of the element.

## Results

### Properties of *Drosophila* eRNA are similar to those observed in vertebrates

Due to the compact nature of the *Drosophila* genome (with an annotated TSS every ∼6.5 kb), many *Drosophila* enhancers reside within genes (either protein coding or noncoding) and in proximity to the 3′ ends of genes. *Drosophila* enhancers therefore are often transcribed as a consequence of the activity of RNA Pol II running through the enhancer due to gene transcription; e.g., lncRNAs or transcriptional read-through at the 3′ ends of genes (Supplemental Fig. S1). To specifically measure transcription initiating at enhancers (eRNA) we used very deeply sequenced CAGE data, providing quantitative information on TSS usage at base-pair resolution (Shiraki et al. 2003; Carninci et al. 2006), and PRO-cap data (Mahat et al. 2016), measuring 5′ ends of short nascent RNA that are still transcriptionally engaged with Pol II, including nascent unstable transcripts. The sensitivity and accuracy of both techniques to detect eRNA has been demonstrated in a number of studies in vertebrates (Andersson et al. 2014a; Core et al. 2014; Arner et al. 2015). We performed strand-specific PRO-cap on tightly staged embryos at two embryonic time windows: 3–4 h after egg laying (AEL), corresponding mainly to blastoderm embryos when the majority of cells is multipotent (∼66 million mapped reads) (Supplemental Table S1), and 6–8 h AEL, mainly stages 10–11, when the major lineages within the mesoderm and neuronal primordia are specified (∼52 million mapped reads) (Supplemental Fig. S2; Supplemental Table S2). This was complemented by our previously published CAGE data (Schor et al. 2017), performed on 81 wild-type inbred lines from the same population at the same two stages of embryogenesis (Materials and Methods). As differences in gene expression are higher between developmental time points than between individuals at a given time point (Cannavò et al. 2017), we combined CAGE reads from all samples at equivalent time points, providing information on transcriptional initiation events at unprecedented depth (1045 million mapped reads) at these two stages of embryogenesis (Supplemental Tables S3, S4).

**Figure 1.**
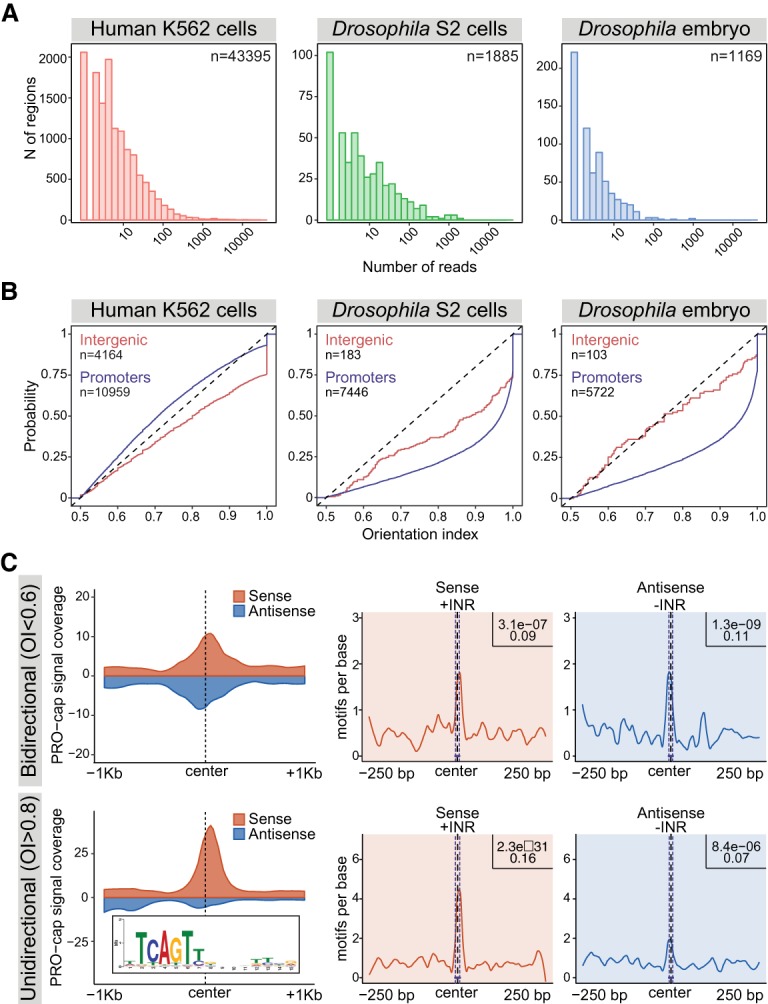
General properties of eRNA are similar between *Drosophila* and vertebrates. (*A*) Histograms of GRO-cap (human K562 cells [Core et al. 2014]) and PRO-cap (*Drosophila* S2 cells [Kwak et al. 2013] and *Drosophila* embryos [6–8 h]) signal at intergenic DHS regions located at least 0.5 and 1.5 kb away from gene 5′ and 3′ ends, respectively. Unannotated TSSs were removed in *Drosophila*. (*B*) Cumulative distributions of transcription orientation index (OI) values estimated (using subsampled data from *A*) over intergenic and promoter DHSs in human K562 cells (Core et al. 2014), *Drosophila* S2 cells (Kwak et al. 2013), and *Drosophila* embryos (6–8 h). OI was estimated as the maximum PRO-cap signal (>500 base pairs [bp] around enhancer center) on either DNA strand divided by the sum of signal from both strands, giving a range between 0.5 and 1 (Core et al. 2012). While *Drosophila* promoters (blue line in S2 cells and embryos) are very directional, intergenic DHSs (yellow lines) have similar bidirectional transcription in both flies and human. (*C*) Levels of eRNA transcription (PRO-cap; 6–8 h), using loess-smoothed summed reads within intergenic DHSs (Supplemental Table S5) centered on the DHS signal maximum. (*Top*) Bidirectional (OI ≤ 0.6; *n* = 456) transcribed intergenic DHSs. (*Bottom*) Unidirectional (OI ≥ 0.8; *n* = 696) transcribed intergenic DHSs (putative enhancers). Sense strand (red) is defined as a strand with a higher transcription level. INR enrichment (using the position weight matrix at the *bottom left*) at the site of maximal transcription initiation in the same regions calculated separately for the sense (red) and antisense (blue) strands. Five-hundred bases around the center are shown; central enrichment and significance were estimated by CentriMo (Bailey and Machanick 2012).

Mammalian eRNAs generally have low abundance, are bidirectionally transcribed, and initiate within regions enriched in the INR motif (Kim et al. 2010; Kaikkonen et al. 2013; Andersson et al. 2014a). To assess these properties in *Drosophila*, we first estimated the relative abundance of eRNA using measurements of nascent transcription in both species: The levels of PRO-cap signal in *Drosophila* embryos (this study) was compared with published GRO-cap (a global run-on sequencing variant) signal in human K562 cells (Core et al. 2014) and PRO-cap signal in *Drosophila* S2 cells (Kwak et al. 2013). To make the analysis comparable between species, we sampled the transcription data so that the median counts at gene promoters (TSS-proximal regions) were similar (Materials and Methods; Supplemental Fig. S3) and estimated transcript abundance at intergenic DNase 1 hypersensitivity sites (DHSs) (John et al. 2011). This comparison indicates that the general distribution of enhancer transcription is comparable between *Drosophila* and humans (Fig. 1A).

We next compared the directionality of *Drosophila* eRNA. In contrast to *Drosophila* promoters, which are generally directionally transcribed, transcription at putative enhancers in S2 cells appears more bidirectional (Core et al. 2012). We reassessed this using our embryonic PRO-cap data and calculated the orientation index (OI) (Core et al. 2012) of intergenic regulatory elements (putative enhancers) in both *Drosophila* and human cells (Supplemental Table S5). The OI represents the fraction of total reads transcribed on the strand with the highest PRO-cap signal (Core et al. 2012). To avoid confounding effects from gene transcription, we divided DHS elements into intergenic (located at least 0.5 and 1.5 kb away from gene 5′ and 3′ ends, respectively; putative enhancers), intragenic, and promoters (overlapping annotated TSS). The intragenic and intergenic elements were further filtered to remove unannotated promoters using genome-wide annotation of lncRNAs (Young et al. 2012) and RAMPAGE-defined TSSs (Batut et al. 2012). Transcription initiating from *Drosophila* promoters, as seen by the cumulative distributions of the OI, is highly directional compared with human promoters, being skewed toward one of the DNA strands (Fig. 1B), an inherent characteristic of fly promoters (Kutach and Kadonaga 2000; Ohler 2006; Core et al. 2012). In contrast, intergenic *Drosophila* elements (putative enhancers) are transcribed with no particular directionality preference, similar to what is observed in mammals (Fig. 1B). We note that while this trend holds true globally, intergenic elements have a continuum of directionality, with some (putative) enhancers displaying bidirectional transcription that is asymmetrically biased to one strand or unidirectional transcription (44% of intergenic elements have an OI > 0.8). This property is also observed in mammals (Ho et al. 2006; Kim et al. 2007; De Santa et al. 2010; Koch et al. 2011) and is one that we explore in more detail below.

*Drosophila* eRNAs transcribed from intergenic DHSs are also associated with INR motifs (Fig. 1C). We examined bidirectional (OI < 0.6) and unidirectional (OI > 0.8) transcribed intergenic DHSs separately for INR motif enrichment (using the position weight matrix [PWM] shown in Fig. 1C). This revealed that the INR motif is positionally enriched at the maximal point of strand-specific eRNA transcription (Fig. 1C). Interestingly, bidirectionally transcribed elements have the INR motif enriched on both strands, while unidirectionally transcribed elements are preferentially enriched on the strand that is more transcribed (Fig. 1C). Taken together, this suggests that the presence (and quality) of one or more INR motifs dictates the mode of enhancer transcription (either unimetrically, bimetrically, or asymmetrically transcribed). In contrast to the INR motif, we observed no enrichment of motifs associated with promoter directionality in mammals (such as the downstream U1 splice sites) or depletion of upstream polyadenylation sites (PASs) (Almada et al. 2013) at putative enhancers (Supplemental Fig. S4), while these motifs have the expected distribution at promoters (Supplemental Fig. S4).

Taken together, these three lines of evidence—namely, the similarity in eRNA levels, their directionality, and positional enrichment of the INR motif—indicate that the general properties of enhancer transcription are conserved between *Drosophila* and vertebrates, strongly suggesting that they arise from a similar mechanism. Given that eRNAs have also been observed in worms (Chen et al. 2013), they are likely a common feature of enhancers across higher metazoa.

### Linking enhancer transcription to enhancer activity during embryogenesis

eRNA transcription is highly correlated with enhancer activity and induced gene expression in vertebrates, as seen in a number of studies in both mice and humans (Plant et al. 2001; Vernimmen et al. 2007; Kim et al. 2010; Wang et al. 2011; Hah et al. 2013; Kaikkonen et al. 2013; Melo et al. 2013). Given this, we next investigated the direct relationship between eRNA and enhancer activity during *Drosophila* embryogenesis, making use of the thousands of characterized developmental enhancers whose spatial and temporal activity has been assessed in vivo in transgenic embryos (Gallo et al. 2011; Bonn et al. 2012a; Kvon et al. 2014). Both PRO-cap and CAGE signals were used as two independent measures of eRNA expression. We compiled a unified set of embryonic enhancers (Materials and Methods), which are either active or inactive during the studied two time windows of embryogenesis. As many of the characterized enhancer regions are quite large (2 kb or more), DHS peaks (Thomas et al. 2010) during embryogenesis were used to refine the enhancer boundaries and remove elements with multiple DHS peaks, which likely span multiple enhancers (Materials and Methods). We also excluded regions within 500 base pairs (bp) of a TSS, both annotated and unannotated (using RAMPAGE data [Batut et al. 2012] and putative lncRNA promoters [Young et al. 2012]). This resulted in a high-stringency set of 1037 embryonic enhancers, each with a single DHS peak and characterized spatio–temporal activity, of which 220 were intergenic (Supplemental Table S6). In addition, we included 63 regions that do not function as enhancers (at least within the tested context) at any stage of embryogenesis (Kvon et al. 2014), referred to as nonenhancers (Supplemental Table S6).

**Figure 2.**
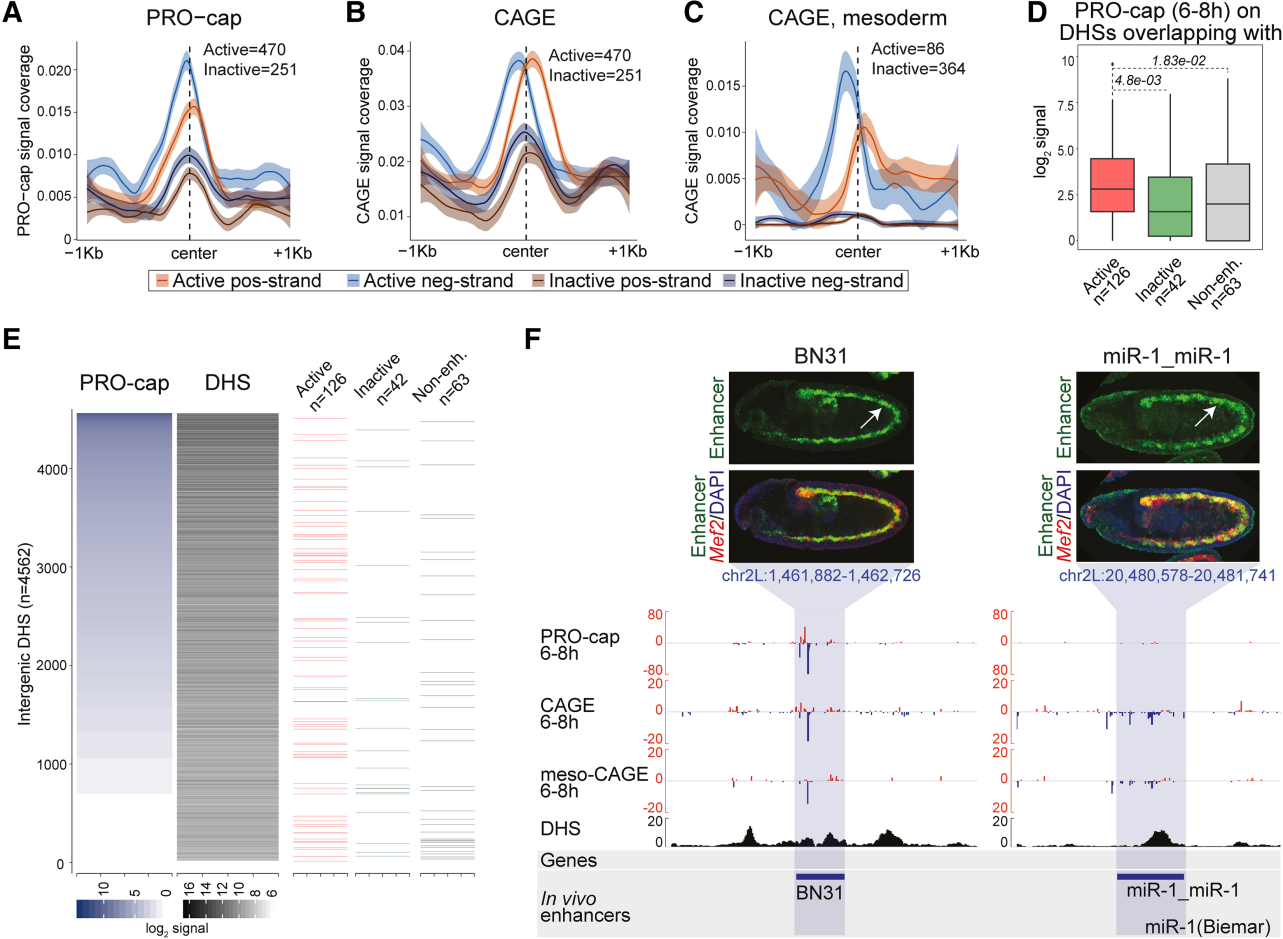
eRNA transcription is correlated with developmental enhancer activity. (*A*–*C*) Levels of eRNA transcription centered on DHSs within characterized developmental enhancers (both intergenic and intragenic) in an active or inactive state. (*A*,*B*) PRO-cap and CAGE (Schor et al. 2017) signal from embryos at 6–8 h at enhancers that are active at 6–8 h in any embryonic tissue or inactive at 6–8 h but active at other time points in any tissue. (*C*) CAGE signal from mesodermal cells (CAGE mesoderm) at 6–8 h at enhancers active in mesoderm at 6–8 h or inactive in mesoderm at 6–8 h but active in other tissues at the same time. (*D*) Box plots show levels of eRNA transcripiton (log_2_, PRO-cap) at 6–8 h in intergenic DHSs within active characterized enhancers (red), inactive enhancers at 6–8 h (green), and nonenhancer regions (gray). *P*-values are from one-sided Wilcoxon rank sum test. (*E*) Heat map showing ranked eRNA signal (PRO-cap; 6–8 h) and corresponding DHS signal (6–8 h; log_2_ of the sum of reads per region) over all intergenic DHSs (*n* = 4562) (Supplemental Table S5). The positions of intergenic enhancers active (126) or inactive (42) at 6–8 h and nonenhancers (63) are shown. (*F*, *top*) Transgenic embryos showing in situ hybridization against the *lacZ* reporter gene (green) and a mesodermal marker (*Mef2*; red). (*Bottom*) Genomic regions showing PRO-cap, CAGE, and mesodermal CAGE (meso-CAGE) signal at 6–8 h on positive strand (red), negative strand (blue), and DNase (black) stage 11 (Thomas et al. 2010). The tested enhancer boundaries are indicated by the horizontal blue shading. The *BN31* enhancer has relatively high levels of transcription (mainly on the negative strand) compared with *miR-1*_miR-1.

Comparing the quantitative levels of eRNA signal with enhancer activity revealed that active enhancers (elements with enhancer activity in transgenic embryos at 6–8 h of embryogenesis) have significantly higher levels of PRO-cap and CAGE signal compared with inactive enhancers (enhancers that are inactive at 6–8 h of embryogenesis but active in transgenic embryos at earlier or later time points) (Fig. 2A,B). Similarly, at early stages of embryogenesis, enhancers that are active at 3–4 h have significantly higher levels of eRNA compared with enhancers that are inactive at these stages but will become active at later embryonic time points (Supplemental Fig. S5A,B). Developmental enhancer transcription in *Drosophila* therefore is highly correlated with the timing of the enhancer's activity. To confirm that eRNA expression also matches the specific tissue in which the enhancer is active, we dissociated 6- to 8-h embryos and sorted mesodermal cells using FACS to >95% purity and performed CAGE on the isolated RNA (Supplemental Fig. S2B; Supplemental Table S7). The level of mesoderm-specific eRNA transcription (quantitative CAGE signal) is significantly higher at enhancers that are active in the mesoderm at 6–8 h compared with enhancers that are inactive in the mesoderm but active in other tissues at these embryonic stages (Fig. 2C). eRNA levels therefore are highly correlated with developmental enhancer activity both temporally and spatially in *Drosophila*, in keeping with similar observations in vertebrate tissues and cell culture models (Andersson et al. 2014a; Wu et al. 2014; Arner et al. 2015).

While this holds true generally, we noticed that within a given tissue and time point, the levels of eRNA are highly varied among different active enhancers. To investigate this further, we ranked all intergenic non-TSS DHS elements at 6–8 h (4562) by their level of PRO-cap signal (Supplemental Table S5) and then visualized where active enhancers (126), inactive enhancers (42), and nonenhancers (63) are located along this quantitative spectrum (Fig. 2E). This revealed that active enhancers have a wide range of eRNA expression, including some that interestingly show no detectable levels of transcription (Fig. 2D,E). Although this “absence of evidence” could be a sensitivity issue, it suggests that not all active enhancers have eRNA. Certainly, not all active enhancers have the same levels of eRNA. To explore this further, we selected two enhancers with very different levels of eRNA and assessed their activity in vivo by linking them to the same minimal promoter and inserting them into the same genomic location in a transgenic reporter assay (Fig. 2F). While both enhancers drive reporter gene expression in the mesoderm at stages 10–11 (6–8 h) to comparable levels, the BN31 (Bonn et al. 2012a) enhancer has much higher eRNA levels compared with the miR1_miR1 (Biemar et al. 2005) enhancer (Fig. 2F). This observed difference in eRNA production between the two enhancers therefore is not due to differences in the numbers of cells in which the enhancers are active; if anything, the miR1_miR1 enhancer (with low eRNA levels) is active in a larger fraction of cells (Fig. 2F). In line with this, some inactive enhancers also transcribe eRNA, as do, surprisingly, some “nonenhancer” regions (Fig. 2E; Kvon et al. 2014). The latter group has no detectable function as developmental enhancers in vivo at any stage of embryogenesis (Kvon et al. 2014) and yet is actively transcribed and DNase-hypersensitive, indicating that it is bound by TFs.

In summary, these results demonstrate a strong correlation between eRNA and enhancer activity, a feature similar to mammals. When transcription is initiating at an enhancer, the enhancer is very likely to be active; however, the converse does not always hold true. Transcription cannot be detected at all active enhancers, suggesting that at least for this subset, eRNA is not mechanistically required for their activity. While enhancer transcription is also highly correlated with enhancer activity in mammals, we note that Andersson et al. (2014a) found that 20%–33% of nontranscribed regulatory regions can activate transcription in an in vitro enhancer assay, suggesting that they may also function as enhancers without eRNA transcription.

### Highly transcribed developmental enhancers have weak promoter activity in vivo

Transcribed enhancers have some promoter-like motifs, including the INR motif, leading to the proposal of a “unified architecture” between enhancer and promoter sequences (Core et al. 2014; Andersson et al. 2015). However, promoters differ strongly from enhancers in their ability to be activated by enhancers (enhancer responsiveness) (Arnold et al. 2017) and to regulate spatial activity. While core promoters have ubiquitous activity, developmental enhancers generally drive specific temporal and spatial expression. We therefore asked whether enhancers with different transcriptional properties (such as transcription orientation, RNA abundance, and stability) have different functional properties in an in vivo promoter assay.

The majority of studies investigating common functional properties between promoters and enhancers (Nguyen et al. 2016; Dao et al. 2017; Diao et al. 2017; van Arensbergen et al. 2017) has used cell culture-based approaches, relying on transient transfection that can be performed at a large scale. However, these assays function outside of a normal chromatinized context, an important property known to affect both promoter (transcriptional initiation) (Archer et al. 1992) and enhancer (Inoue et al. 2017) activity through various mechanisms (Jeong and Stein 1994; Hebbar and Archer 2008). We therefore set out to assess the ability of enhancers to act as promoters in vivo in transgenic embryos using a stably integrated reporter that does not contain a core promoter.

**Figure 3.**
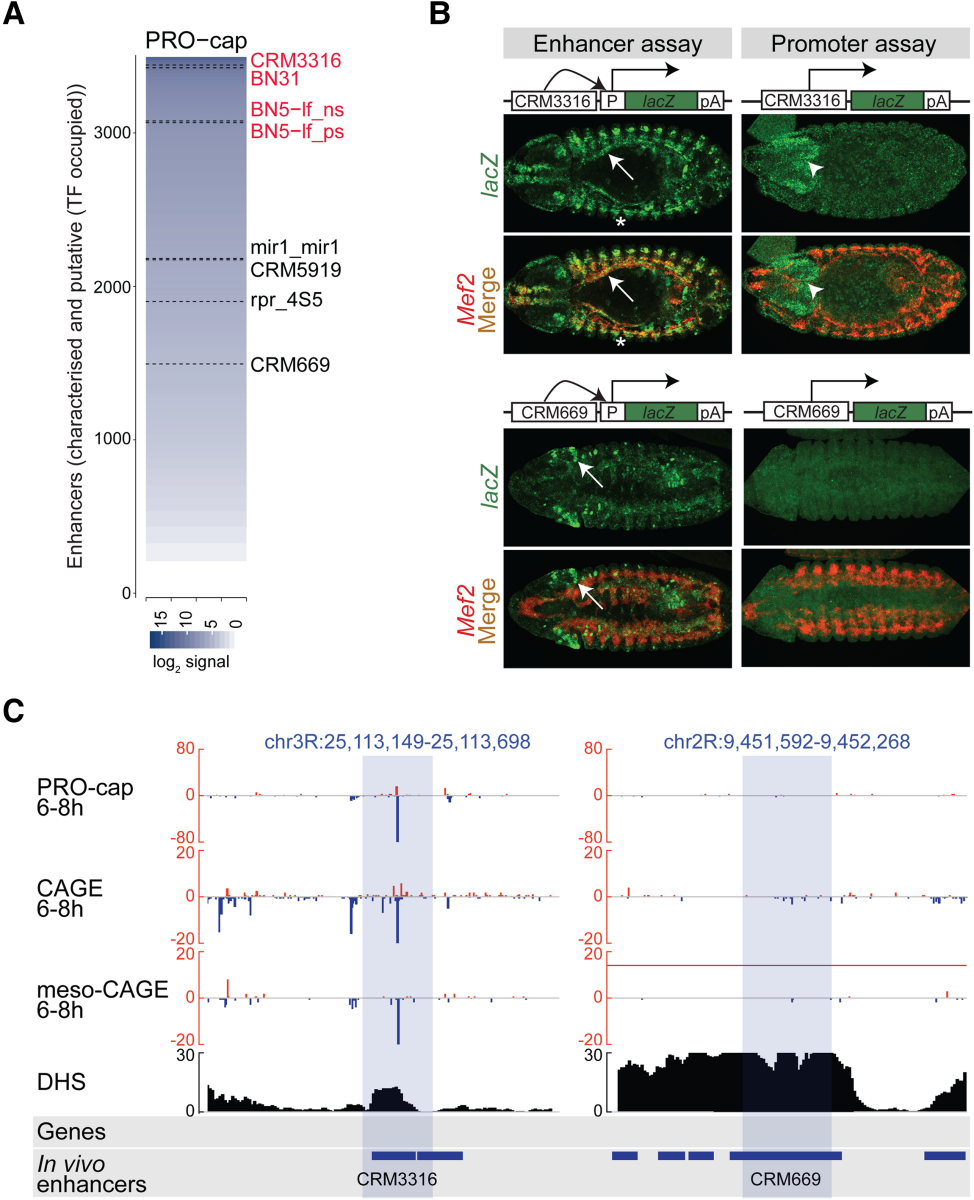
Highly transcribed enhancers can function as promoters in vivo. (*A*) Ranked PRO-cap 6- to 8-h signal (log_2_) at characterized and putative enhancers based on mesodermal TF occupancy (Zinzen et al. 2009), both intergenic and intragenic (*n* = 3492) (Supplemental Table S8). Eight regions tested for enhancer and promoter activities in vivo are indicated with high (red) or low (black) transcription. (*B*) Double in situ hybridization of transgenic embryos with probes directed against the *lacZ* reporter gene driven by the tested element (green) and mesoderm marker gene *Mef2* (red). (*Top* panel) *CRM3316* acts as an enhancer, driving expression in the somatic (asterisk) and visceral (white arrow) muscle and overlapping *Mef2*. Expression driven by *CRM3316* in the promoter assay is localized to the brain (white arrowhead). Embryos (stage 13) are ventrally oriented with anterior to the left. (*Bottom* panel) *CRM669* acts as an enhancer, driving expression in the head ectoderm (white arrow), but has no detectable activity as a promoter. Embryos (stage 14) are dorsally oriented with anterior to the left. (*C*) Genomic loci of *CRM3316* and *CRM669* showing PRO-cap, CAGE, and meso-CAGE (from mesodermal cells sorted by FACS) signal at 6–8 h on both the positive (red) and negative (blue) strands. DHSs at stage 11 (spanning 6–8 h) (from Thomas et al. 2010). Blue shading indicates the boundaries of tested regions.

We first ranked enhancers–both in vivo characterized (Gallo et al. 2011; Bonn et al. 2012a; Kvon et al. 2014) and predicted (Zinzen et al. 2009)—by their levels of eRNA based on their nascent PRO-cap signal (Fig. 3A; Supplemental Table S8). We selected eight elements: four with high (arbitrary cutoff >240 PRO-cap reads) and four with low (at least four times lower; <60 PRO-cap reads) levels of eRNA. Each element was inspected manually to confirm that no other source of transcription was located in their proximity, which could confound the eRNA signal (Fig. 3C). All eight elements were placed in front of a *LacZ* reporter with and without a minimal *hsp70* promoter to examine their ability to function as an enhancer or promoter, respectively (Fig. 3B; Supplemental Fig. S6). Five of these regions were shown previously to function as an enhancer in vivo (Lohmann 2003; Biemar et al. 2005; Bonn et al. 2012a). Here, the cloned candidate regions were centered on the maximum eRNA signal (median size 613 bp) (Supplemental Table S9) and inserted in an orientation where the maximum transcription occurred in the direction of the reporter gene. The derived 16 constructs were stably integrated into the same genomic location (51C landing site of the J27 line) using the φC31 integrase system (Bischof et al. 2007) to minimize positional effects. Both enhancer and promoter activities were assessed by fluorescent in situ hybridization (FISH) with a probe directed against the *LacZ* reporter gene.

As expected, seven out of eight elements displayed specific enhancer activity in vivo, the exception being a previously untested element*.* Importantly, promoter activity was detected in four out of eight cases, three of which have high levels (*CRM3316* [Fig. 3B], *BN5-lf-p*, and *BN5-lf-n* [Supplemental Fig. S6]) and one that has low levels (*rpr4s5* [Supplemental Fig. S6]) of endogenous eRNA. This suggests a relationship between the level of eRNA and an enhancer's inherent capacity to act as a promoter in vivo, although we note that the number of tested enhancers is low. In all four cases, the relative intensity of promoter signal was much weaker than that of the enhancer-driven expression, a feature also observed in mammalian cells (Nguyen et al. 2016; van Arensbergen et al. 2017).

For three of the four enhancers, the spatial pattern of their promoter activity partially overlapped that of the enhancers’ activity but was limited to a smaller fraction of cells (e.g., *BN5-lf-*p) (Supplemental Fig. S6). Therefore, when the element is functioning as both an enhancer and a promoter, the TFs that bind to the enhancer are still able to direct Pol II activity in a specific spatial pattern. For the remaining enhancer (*CRM3316*) (Fig. 3B), the spatial pattern of the promoter's activity was different from when the element was functioning as an enhancer (in the presence of the *hsp70* minimal promoter): While *CRM3316* drives enhancer activity in the mesoderm, it regulates promoter activity in the central nervous system (Fig. 3B). This suggests that this element is acting as a promoter for another “external” enhancer, which is likely a neuronal enhancer in the vicinity of the transgenic integration site (discussed further below).

In summary, these results demonstrate that intergenic enhancers can act as weak promoters in vivo. While, in three cases, the element acted as both an enhancer and promoter, driving activity in overlapping patterns, in the fourth case, the enhancer possibly functioned as an ectopic promoter for an external enhancer. We note that not all transcribed enhancers (∼50%) have detectable promoter activity, and this seems to correlate with their levels of eRNA production. In the transgenic assay, promoter activity is measured by in situ hybridization against *lacZ*, a long (4309 bp), stable, polyadenylated mRNA. Therefore, what we are classifying based on RNA sequencing (RNA-seq) methods as “high” and “low” eRNA likely reflects both eRNA stability and inherent promoter strength. Enhancers that can act as promoters in vivo may have stronger inherent promoter sequences (e.g., INR motif) and/or other properties that contribute to transcript stability.

### A dual transgenic assay to measure both enhancer and promoter activity in vivo

**Figure 4.**
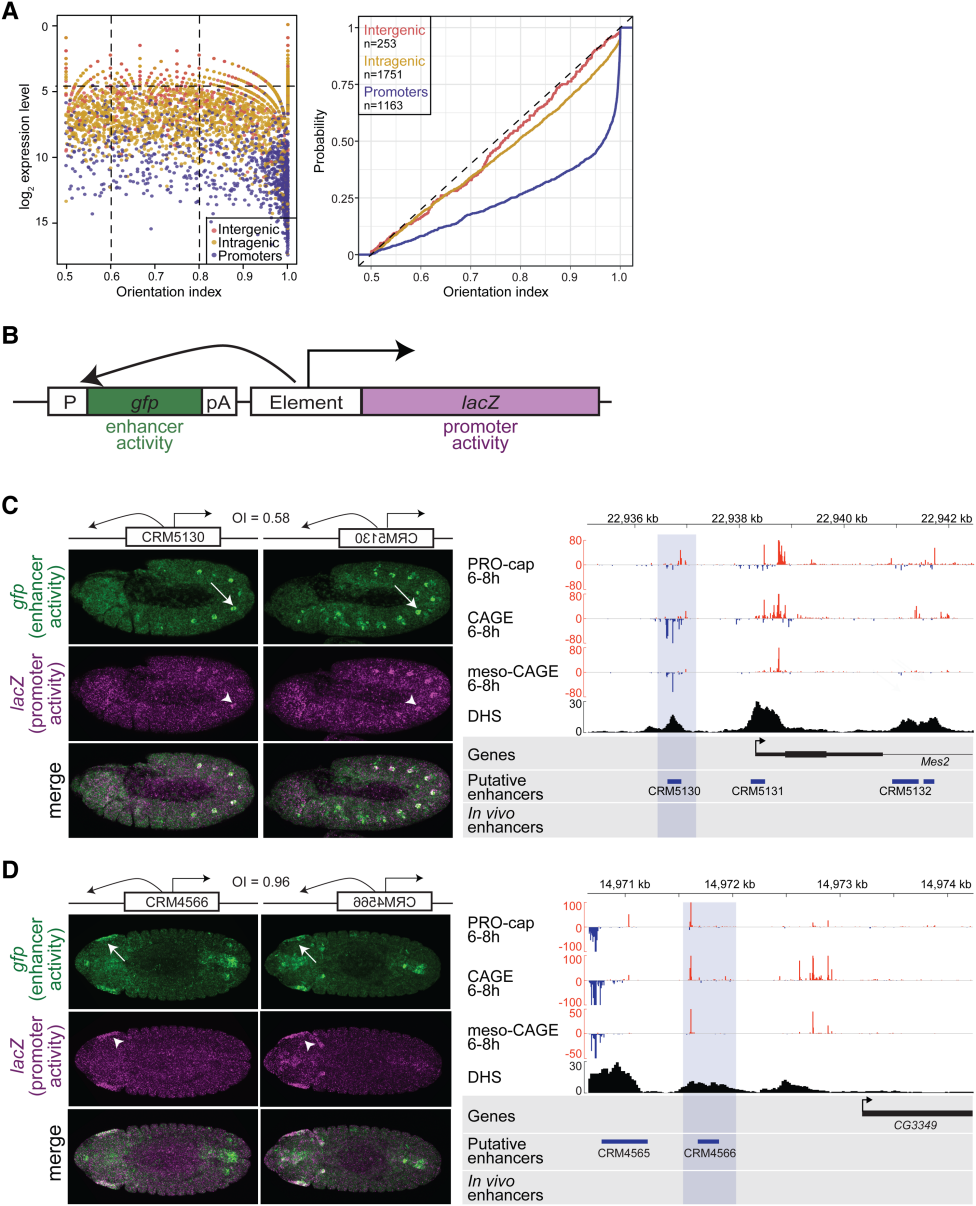
The direction of eRNA trancription is associated with the ability of an enhancer to function as a promoter. (*A*, *left* panel) eRNA expression (*Y*-axis; log_2_ PRO-cap 6–8 h) and the transcription OI (*X*-axis) at intergenic (red; *n* = 253) and intragenic (yellow; *n* = 1751) enhancers and promoter regions (blue; *n* = 1163). Enhancers is a combined set of characterized (active at 6–8 h) and putative (bound by at least one mesodermal TF at 6–8 h) enhancers (Supplemental Table S8; Zinzen et al. 2009). The OI was estimated as the maximum PRO-cap signal (>500 bp around enhancer center) on either DNA strand divided by the sum of signal from both strands, giving a range between 0.5 and 1 (Core et al. 2012). (*Right* panel) The cumulative probability distributions (*Y*-axis) of OI values (*X*-axis) calculated for intergenic (red) and intragenic (yellow) enhancer and promoter regions (blue), as in *A*. To remove outliers, only elements with >30 reads (intergenic, intragenic, and TSS), corresponding to 0.25 quantile (horizontal dashed line in the *left* panel), are plotted. *n* = 3167. Intergenic and intragenic enhancers show a bidirectional transcriptional pattern, while promoter regions are more unidirectionally transcribed. (*B*) Schematic of the dual-reporter vector used to measure enhancer (green) and promoter (magenta) activities simultaneously in the same embryos at the same genomic location. (P) *hsp70* minimal promoter; (pA) polyadenylation signal; (element) putative regulatory element inserted into a multiple cloning site. (*C*,*D*, *left*) Double in situ hybridization against *gfp* driven by the minimal promoter under the control of the inserted element (enhancer activity; green) and the *lacZ* reporter driven by the inserted element (promoter activity; magenta). (*Top*) *CRM5130* (Zinzen et al. 2009) drives highly overlapping enhancer (white arrow) and promoter (white arrowhead) expression in oenocytes and somatic muscle in both sense and antisense orientations. Embryos (stage 11) are laterally oriented with anterior to the left. (*Bottom*) *CRM4566* (Zinzen et al. 2009) has enhancer activity (green) in the hindgut and head ectoderm (white arrow) and acts as promoter (magenta) in the head ectoderm (white arrowhead) in both orientations. Embryos (stage 13) are laterally oriented with anterior to the left. OIs of endogenous transcription: 0.58 bidirectional and 0.96 unidirecitional. (*Right*) Genomic loci of *CRM5130* and *CRM4566*, showing PRO-cap, CAGE, and meso-CAGE (from FACS-sorted mesodermal cells) signal at 6–8 h on both the positive (red) and negative (blue) strands. DHSs at stage 11 (spanning 6–8 h) (from Thomas et al. 2010). Blue shading indicates the boundaries of tested regions.

The standard enhancer reporter constructs used for transgenic studies in both flies and mice place the tested enhancer upstream of a minimal promoter. This includes the widely used *Drosophila pH* Pelican and *pH* Stinger vectors (Barolo et al. 2000) and the modified vector that we used above (Fig. 3B; Supplemental Fig. S6). However, with this arrangement, it is impossible to discriminate between an enhancer's inherent promoter or enhancer activity, as transcription may originate from the minimal promoter or the candidate region inserted directly upstream of it. To address this, we generated a new vector that can simultaneously assess both enhancer and promoter activity from the same element in the same embryo. The dual enhancer–promoter activity vector contains a multiple cloning site in between an upstream *GFP* reporter (containing a minimal promoter) and a downstream *LacZ* (with no minimal promoter) (Fig. 4B). If the regulatory element can act as an enhancer, it should be able to regulate *GFP* expression from a distance, while, if it can act as a promoter, it will regulate *LacZ* expression. To confirm that changing the position of the regulatory element (relative to the minimal promoter) does not affect its enhancer activity, we first directly compared the activity of a characterized enhancer (*BN5-lf-p*) (Bonn et al. 2012a) cloned into the dual enhancer–promoter vector and a modified *pH* Pelican vector, both of which were stably integrated into the same genomic location (Supplemental Fig. S7). As expected, placing the enhancer further away from the target gene's promoter (*GFP*) led to a reduction in the level of gene expression (Fukaya et al. 2016). However, notably, the spatio–temporal pattern of the enhancer's activity in the somatic muscle is the same in both constructs (Supplemental Fig. S7). Importantly, by placing the enhancer downstream from the reporter gene, the enhancer–promoter dual vector can unequivocally distinguish between enhancer and promoter activities.

### Enhancer versus promoter function is reflected in the orientation of eRNA transcription

Enhancers were originally defined as elements that regulate transcription at variable distances from their target genes and in an orientation-independent manner (Banerji et al. 1981; Moreau et al. 1981). In contrast, promoters have directionality in their initiation (Almada et al. 2013), especially in *Drosophila* (Fig. 1B; Core et al. 2012). *Drosophila* intergenic and intragenic enhancers are transcribed both unidirectionally and bidirectionally (Fig. 4A), with a wide range in between where transcription is asymmetrically biased to one strand compared with the other. In light of this, we next assessed whether there is a relationship between the direction of endogenous enhancer transcription and its ability to function as a promoter in vivo. To do this, we changed the orientation of eRNA transcription by flipping the orientation of the regulatory element with respect to the reporter gene. Given our observation that enhancers with “high” eRNA can generally function as promoters (Fig. 3; Supplemental Fig. S6), we focused on enhancers with relatively high levels of eRNA (>22 CAGE counts in at least one orientation) but differences in the directionality in which the eRNA is transcribed. We selected 20 DHS regions overlapping in vivo validated or putative enhancers from TF occupancy (Zinzen et al. 2009) with a range of eRNA OIs: bidirectional (OI < 0.6), unidirectional (OI > 0.8), and those in between (0.6 < OI < 0.8), which are generally asymmetrically biased to transcription on one strand, representing ∼41% of all enhancers (Fig. 4A).

**Figure 5.**
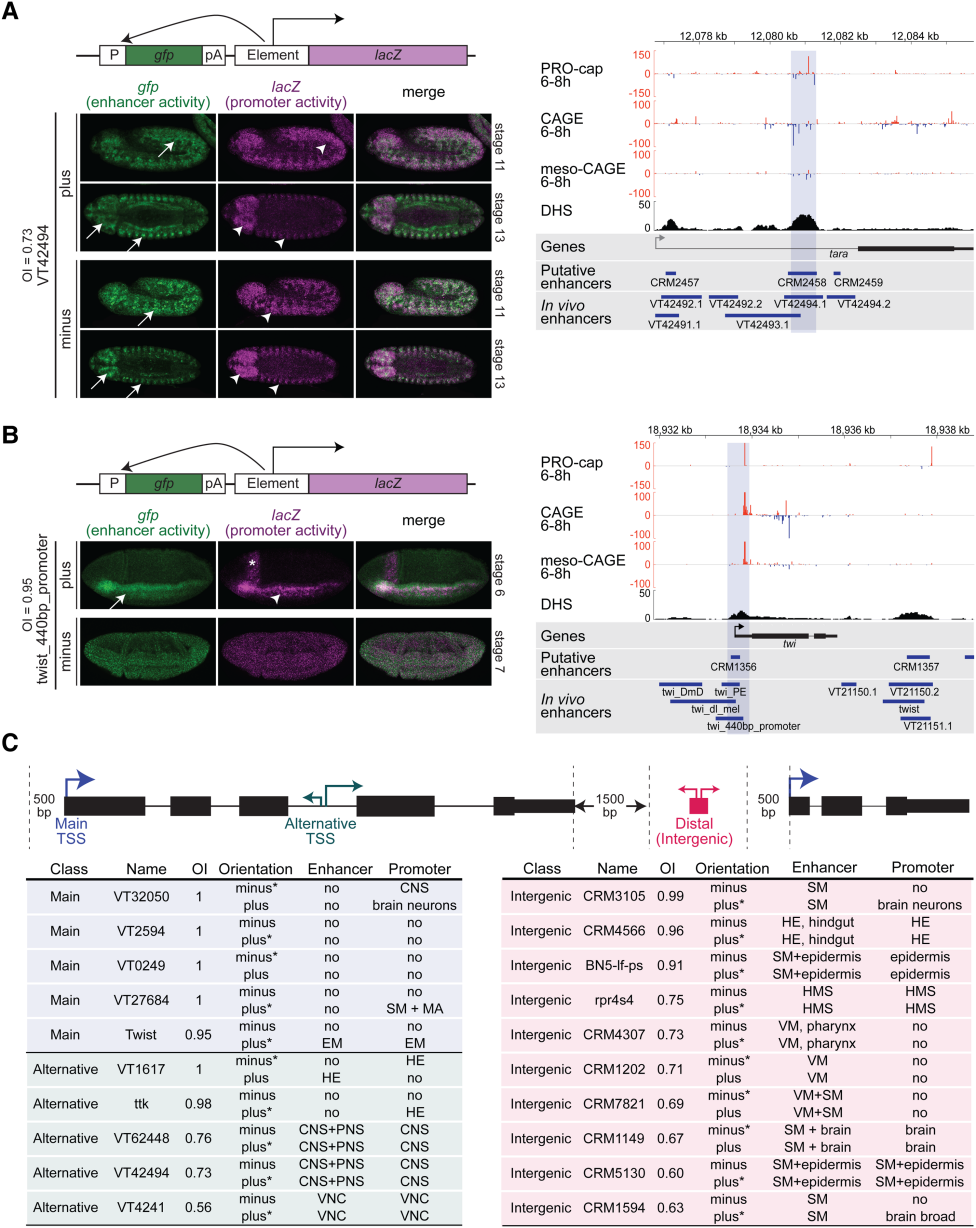
Gene promoters can act as developmental enhancers. (*A*,*B*) Double in situ hybridization against *gfp* driven by the minimal promoter under the control of the inserted element (enhancer activity; green) and the *lacZ* reporter driven by the inserted element (promoter activity; magenta). (*A*, *left*) *VT42494* (Kvon et al. 2014) drives overlapping enhancer (green) and promoter (magenta) expression in the peripheral nervous system and embryonic brain (white arrows, arrowheads) in sense and antisense orientations. Embryos are laterally (stage 11) or ventrally (stage 13) oriented with anterior to the left. (*B*, *left*) The *Twist_440 bp_promoter* element has overlapping enhancer (green) and promoter (magenta) activity in the presumptive mesoderm (arrows) in the sense (plus) orientation. The asterisk indicates the cephalic furrow. No activity was detected in the other orientation for either the enhancer or the promoter. Embryos are laterally (stage 6 or 7) oriented with anterior to the left. (*A*,*B*, *right*) Genomic loci of *VT42494* and *Twist_440 bp_promoter* showing PRO-cap, CAGE, and meso-CAGE (from FACS-sorted mesodermal cells) signal at 6–8 h on both the positive (red) and negative (blue) strands. DHSs at stage 11 (spaning 6–8 h) (from Thomas et al. 2010). Blue shading indicates the boundaries of tested regions. (*C*) Summary of the activity of all of the tested elements in the dual transgenic assay. (Orientation) Plus or minus DNA strand; (*) strand with higher endogenous transcription; (enhancer) tissues where enhancer (*gfp*) activity was detected; (promoter) tissues where promoter (*lacZ*) activity was detected; (CNS) central nervous system; (SM) somatic muscle; (MA) midgut anlage; (HE) head ectoderm; (PNS) peripheral nervous system; (VNC) ventral nerve cord; (HMS) head maxillary segment; (VM) visceral muscle.

Of the 20 tested regions, 10 were intergenic DHSs (including one enhancer that we tested in the standard upstream assay) (Fig. 3), which have a range of eRNA OIs. We also included 10 gene promoters comprised of five alternative TSSs (three with various degrees of bidirectional transcription [OIs of 0.56, 0.73 and 0.76] and two unidirectional [OIs of 1 and 0.98]) and five primary main TSSs as more “extreme” examples of directional elements (OI values between 0.95 and 1) (Fig. 5C). “Main TSSs” were classified as the TSSs with the maximum CAGE signal at 6–8 h, while the remaining were assigned as alternative TSSs (using unambiguous TSS-to-gene assignments from Batut et al. 2012; FlyBase). For each genomic element, the region encompassing the DHS peak (mean size of 487 bp) (Supplemental Table S9) was cloned in both the sense and antisense orientation, leading to 41 transgenic lines, including an empty control line. While enhancer orientation (relative to the reporter gene's promoter) is expected to have no effect on enhancer activity, to our knowledge, the effect of orientation on promoter activity (*LacZ* expression) has not been assessed in vivo.

All intergenic regions tested function as enhancers in vivo, regulating *GFP* expression in specific tissues and stages (Fig. 4C,D; Supplemental Fig. S8). In all 10 cases, the enhancer functioned in both orientations, regulating qualitatively similar spatio–temporal activity (Fig. 4C,D; Supplemental Fig. S8). Notably, seven out of the 10 intergenic enhancers (including one previously tested in the single activity assay [*BN5-lf-p*]) (Supplemental Fig. S7) can also function as promoters in vivo, regulating weak but spatially restricted *LacZ* expression. This suggests that at least a fraction of *Drosophila* enhancers that initiate transcription can function as weak promoters in vivo. We note that as the dynamic range of transcription measured by deep RNA-seq is much higher than that detected by in situ hybridization, we may have missed low levels of promoter activity in our in vivo reporter assay and thereby underestimated this function. In five out of seven cases, the promoter activity at least partially recapitulates the enhancer activity: *CRM5130* (Fig. 4C), *CRM4566* (Fig. 4D), *BN5-lf-p* (Supplemental Fig. S7), *CRM1149*, and *rpr4s4* (Supplemental Fig. S8). In the remaining two cases (*CRM1594* and *CRM3105*) (Supplemental Fig. S8), two somatic and visceral muscle enhancers drive weak promoter expression in the brain, mirroring the result observed for *CRM3316* inserted in the same location (Fig. 3B; Supplemental Fig. S8), suggesting that their promoter activities might be controlled by an external enhancer. Interestingly, both *CRM1594* and *CRM3105* produced this promoter pattern in an orientation-dependent manner (Supplemental Fig. S8). Why only three of the 27 tested elements (20 in the dual assay and eight in the single assay, with one in common) were susceptible to this extrinsic enhancer's activity in the promoter assay is unclear. It may reflect potential enhancer core promoter specificity, as shown in other cases (Butler and Kadonaga 2001; Zabidi et al. 2015). However, given this, we conclude that the spatial promoter activity intrinsic to all of the enhancers tested recapitulates all or a subset of the enhancer activity.

Eight of the 10 tested promoters (five out of five alternative TSSs and three out of five main TSSs) can function as promoters in vivo, as expected (Fig. 5; Supplemental Fig. S9). The majority of tested elements has ∼10-fold higher endogenous transcription in one direction (the direction of the gene's expression) and is therefore highly unidirectional. We used these elements and the 10 intergenic elements, which have a range of bidirectional and unidirectional transcription, to determine whether the orientation of the endogenous eRNA's transcription is predictive of promoter activity. Assessing promoter activity of each element when inserted in both orientations revealed a strong association between the direction of its endogenous transcription and the element's ability to function as a promoter in the in vivo assay. Of the 15 elements that function as promoters, eight had detectable promoter activity in both orientations and corresponded to elements that were endogenously bidirectional yet often asymmetrically transcribed (with variable OIs <0.8, with two exceptions that have largely unidirectional transcription: *CRM4566* [OI = 0.96] and *BN5-lf-p* [OI = 0.89]). In the case of *BN5-lf-p*, the promoter activity appears stronger in the orientation of endogenous transcription (Supplemental Fig. S7), although we note that the in situ hybridizations are not strictly quantitative.

In contrast, seven elements could function only as a promoter in one orientation, and, in all cases, this corresponds to the direction in which the bulk of their endogenous eRNA is transcribed (*Twist_440bp_promoter* [Fig. 5B], *CRM1594*, *CRM3105* [Supplemental Fig. S8], *VT32050*, *VT27684*, *ttk*, and *VT1617* [Supplemental Fig. S9]). Five of these overlap gene promoters that are strongly unidirectionally transcribed, either main or alternative, where the direction of the endogenous transcription is clearly linked to the orientation in which it can function as a promoter.

An overview of all of the elements’ activity and directionality (OI) is shown in Figure 5C. To summarize, the ability of enhancers to initiate transcription in a chromatinized context appears relatively common. Our results demonstrate that endogenous promoter and enhancer activities can coexist within the same element to regulate similar spatio–temporal activity during embryonic development and suggest a relationship between the direction of endogenous transcription and the orientation in which the element can function as a promoter in vivo. The only three exceptions (out of 15) are intergenic enhancers that have either unidirectional transcription but act as a promoter in both orientations (*CRM4566* [Fig. 4D] and *BN5-if-p* [Supplemental Fig. S7B]) or bidirectional (although asymmetric; OI = 0.68) transcription that can only function as a weak promoter in one orientation (*CRM1594*) (Supplemental Fig. S8).

### Alternative gene promoters can function as developmental enhancers in vivo

A number of recent studies using large-scale cell culture assays indicate that a fraction of promoters can act as enhancers (Arnold et al. 2013; Zabidi et al. 2015; Nguyen et al. 2016; Dao et al. 2017; Diao et al. 2017). Here, we investigated whether this also holds true in a chromatinized background during embryonic development. Out of the 10 gene promoter regions tested above (five main TSSs and five alternative TSSs), eight overlapped a 2-kb region that was reported previously to have enhancer activity, and two (*VT2049* and *VT1617*) were reported to have no enhancer activity (Kvon et al. 2014) and therefore served as negative controls. Previously, these 2-kb fragments were placed upstream of a minimal promoter and reporter (Kvon et al. 2014), as in standard enhancer transgenic assays. Here we cloned ∼400-bp single DHS regions encompassing the promoter, placed them in the dual enhancer–promoter vector, and assessed the ability of these promoter regions to also function as developmental enhancers.

In five out of 10 cases, the tested promoter region can function as a developmental enhancer in vivo. Interestingly, four of these five regions (*VT4241*, *VT42494*, *VT62448*, and *VT1617*) are alternative TSSs; in all four cases, the elements functioned as both a promoter and an enhancer, giving largely overlapping spatial patterns of activity (Fig. 5A; Supplemental Fig. S9). In three cases, both promoter and enhancer activities occurred in both orientations, in keeping with the bidirectional transcription of these elements (although asymmetric: OI values at 6–8 h for *VT4241*, *VT42494*, and *VT62448* were 0.7, 0.72, and 0.78, respectively) (Fig. 5A, summarized in C; Supplemental Fig. S9). In contrast, the fourth region overlapping an alternative TSS, *VT1617*, has orientation-dependent activity and unidirectional transcription. This element does not have enhancer activity when tested in one orientation (Supplemental Fig. S9B), as observed previously (Kvon et al. 2014). However, it does have enhancer activity in the head at early stages of embryogenesis in the other orientation in addition to orientation-dependent promoter activity (Supplemental Fig. S9B). This region has very strong unidirectional transcription (>4500-fold higher on the antisense strand) (Supplemental Table S10), in keeping with its directional ability to function as a promoter. However, this orientation dependence for the enhancer activity is surprising. The enhancer and promoter spatial patterns driven by this element are very similar, although, intriguingly, the two activities were observed in the opposite orientations.

Interestingly, none of the main TSSs possesses enhancer activity, with the exception of the *twist* gene (Fig. 5B,C; Supplemental Fig. S9). The tested region contains a single DHS that spans the gene's main promoter and a characterized enhancer—the Twist promoter-proximal element (*Twist_440 bp_promoter*)—that is located in very close proximity to the main *twist* TSS (Jiang et al. 1991). This element functions as an enhancer, as reported previously, but, surprisingly, our results indicate that it does so in only one orientation (Fig. 5B, plus): when positioned toward the *lacZ* gene in the same orientation as its unidirectional transcription toward the endogenous *twist* gene (Fig. 5B, plus strand). Moreover, the spatial activity of this element when functioning as a promoter is broader than its spatial enhancer activity, being active also in the cephalic furrow (Fig. 5B, asterisk). In the other orientation, where its unidirectional transcription extends antisense toward *gfp*, the element had no detectable enhancer activity (i.e., sense *gfp* transcription). This orientation-dependent enhancer activity may be due to transcriptional interference from antisense *gfp* transcription, although we note that there was no detectable antisense *gfp* transcription by in situ hybridization (data not shown).

Taken together, these results indicate that some elements, either TSS-overlapping or TSS-proximal, can function as both a promoter and an enhancer (summarized in Fig. 5C). This feature seems to be conserved in vertebrates (Dao et al. 2017). In our case, the enhancer activity overlaps the spatial expression of the associated gene, suggesting that the element is enhancing the expression of the same gene in which it acts as a promoter. Our results indicate a strong correlation between the direction of endogenous promoter transcription and the ability of the element to function as an orientation-independent enhancer. Strictly unidirectional promoters had orientation-dependent enhancer activity, while only bidirectionally transcribed promoters (OI < 0.8) had orientation-independent enhancer activity.

## Discussion

Through the integration of information on transcription initiation in the noncoding genome (using deeply sequenced CAGE and PRO-cap) with that of developmental enhancer activity (using hundreds of in vivo characterized embryonic enhancers), we assessed the general properties of *Drosophila* eRNA. Our results indicate that the general features of eRNA are highly conserved from flies to humans, including the level and orientation of eRNA transcription and the relative positioning of the INR motif. During the course of this study, we generated 56 transgenic lines to functionally assess regulatory elements with different eRNA properties for both enhancer and promoter activity. Our results uncovered a number of intriguing features suggesting that there is a continuum of enhancer and promoter functions matching the continuum of endogenous transcription.

### eRNA levels generally correlate with enhancer activity

Comparing endogenous enhancer transcription with endogenous enhancer activity in transgenic embryos revealed a very strong global correlation between both the timing (developmental stage) and place (tissue) of enhancer activity. This is consistent with similar global comparisons in cell culture models and suggests a mechanistic link to TF occupancy or some other property of enhancer function. However, we also observed that active enhancers have a wide range of eRNA levels, with many active enhancers having very low or undetectable eRNA at the stages when the enhancer is active. Similarly low levels of eRNA may also occur in other species; 35% of putative *C. elegans* enhancers do not overlap transcription initiation clusters (TICs) (Chen et al. 2013), while 60% of intergenic putative mouse enhancers do not contain eRNA, as reported in one study (Kim et al. 2010). While these percentages may be overestimated due to the inclusion of elements that are not enhancers, nearly a third (20%–33%) of nontranscribed regulatory regions demonstrated enhancer activity in a luciferase assay (Andersson et al. 2014a). In the context of our study, all elements were confirmed embryonic enhancers, and we carefully matched the stage of enhancer activity to the stage of eRNA detection. Active embryonic enhancers therefore are transcribed in a broad range, with the highly transcribing enhancers producing several orders of magnitude more transcripts than those with the weakest transcription, suggesting that eRNA production and enhancer activation can be uncoupled (at least for a subset of enhancers). For enhancers with very weak transcription, eRNAs are likely to be present only sporadically or in a minority of cells, suggesting that their continued presence is unlikely to be essential for these enhancers’ function, although the act of transcription might be.

### Intergenic embryonic enhancers can function as weak promoters

The presence of Pol II and the basal transcriptional machinery at enhancers (Koch et al. 2011) and their ability to transcribe eRNAs question whether there is an inherent difference between an enhancer and a promoter, with some proposing a unified architecture between the two (Andersson et al. 2015). To disentangle both activities, we developed a new dual transgenic assay that can measure enhancer and promoter activity at the same genomic location in the same embryos such that the timing as well as tissue specificity of both activities can be directly compared. Transgenic assays have the advantage of being able to measure regulatory activity at the endogenous levels of TFs and within a consistent chromatinized context—two properties that have a major impact on both enhancer and promoter activity (Archer et al. 1992; Jeong and Stein 1994; Hebbar and Archer 2008; Inoue et al. 2017). The readout (in situ hybridization) provides both spatial and temporal information at single-cell resolution, although it is difficult to derive quantitative information on activity—a clear disadvantage compared with in vitro reporter assays.

We tested 27 regulatory elements (20 in both orientations) from different genomic locations and with different transcriptional properties for both enhancer and promoter activity. Our results indicate that highly transcribed developmental enhancers can function as weak promoters in vivo. The spatial pattern of promoter activity was generally a subset of the tissues in which the enhancer was active, indicating that both activities can occur in the same cells from the same element. This promoter function depended largely on the orientation in which the element was inserted, matching the direction of enhancer transcription in its endogenous location: Bidirectional elements (both enhancers and promoters) can generally function as promoters in both orientations, while unidirectional elements have orientation-dependent activity (Fig. 5C). This indicates that promoter activity has intrinsic directionality and suggests the presence of directional sequence motifs within enhancer elements. In keeping with this, bidirectional mammalian promoter regions contain separate motifs that promote transcription in either direction (Core et al. 2014; Duttke et al. 2015); our results point to a similar sequence-based determinant of enhancer directionality in *Drosophila,* supported by the presence of potential “pairs” of INR motifs within bidirectional enhancers at the two points of maximal divergent transcription (Fig. 1C). Intragenic enhancers have been shown previously to act as alternative promoters, regulating unidirectional transcription in the direction of the host gene's expression to produce lncRNAs that are abundant, stable (polyadenylated), and spliced (Kowalczyk et al. 2012). In the case of the intergenic enhancers studied here, we have no evidence that they produce stable long transcripts. Standard strand-specific poly(A)^+^ RNA-seq did not detect any RNA at the vast majority of these enhancers, suggesting that Pol II elongation is fundamentally different at intergenic versus intragenic enhancers.

### A subset of promoters can function as developmental enhancers

Recent high-throughput studies indicate that the same sequences can function as both promoters and enhancers in vitro, although gene promoters generated more promoter activity compared with distal elements (Nguyen et al. 2016; van Arensbergen et al. 2017). While our results also show that the same sequences can harbor both activities, we uncovered some key differences. Tested elements that overlap a gene's main promoter, while acting as strong promoters both endogenously and in the promoter assay, do not possess enhancer activity (at least for four of the five elements examined). In contrast, some alternative gene promoters have an intriguing dual functionality, being able to act with seemingly equal strength as strong enhancers and promoters at the same stage in the same tissues. Using luciferase assays, Li et al. (2012) found that strong and weak promoters have different enhancer activities with an inverse relationship between the two functions. Here, in the context of embryonic development, our results generally agree with this: Strong promoters (the main genes’ promoters) generally have no detectable enhancer activity, while “strong” (highly active) intergenic enhancers have weak (or not detectable) promoter activity (at least for the ones that we tested). This indicates that developmental enhancers and gene promoters generally have different intrinsic properties.

However, we also found interesting intermediate cases between the two, which suggests a relationship between the directionality of eRNA transcription and the ability to function as an enhancer or promoter in vivo. When bidirectionally transcribed, alternative gene promoters can function as both strong promoters and enhancers in vivo in both orientations (Fig. 5A,C). In contrast, when unidirectionally transcribed, the element can generally function only as a promoter (and, in a few cases, as an enhancer) in an orientation-dependent manner matching its direction of transcription. One interesting example of the latter is an ∼400-bp DHS element that overlaps the promoter of the *twist* gene and is transcribed in a unidirectional manner. We show that this element can function as both a promoter and an enhancer but, interestingly, can perform both functions in only one orientation and is inactive in the other (Fig. 5B). These results suggest that some enhancers may have evolved to drive proximal orientation-dependent activation, possessing strong intrinsic promoter potential but lacking the ability to act more distally in an orientation-independent manner. To summarize, bidirectional *cis*-regulatory elements (either enhancers or promoters) can often function as both enhancers and promoters (although to different degrees) in an orientation-independent manner. In contrast, unidirectional elements generally function only in the orientation in which they are transcribed.

**Figure 6.**
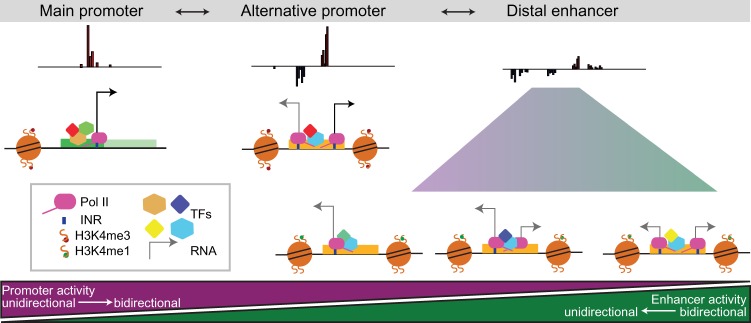
A continuum of eRNA directionality and levels reflects *cis*-regualtory function. Schematic representation of gene promoters, alternative gene promoters, and enhancers showing the heterogeneity of transcription directionality (arrowhead orientation), abundance (arrow height), and stability (arrow thickness) across regulatory elements. Going from *left* (strict promoters) to *right* (strict enhancers), the levels and orientation of transcription vary, as do the functional properties of the *cis*-regulatory elelments, going from highly directional promoters to nondirectional enhancers, with intermediate combinations with dual functionality in between.

Taken together, our results suggest a continuum of functions that mirrors the continuum of eRNA directionality and levels of transcription at *cis*-regulatory elements (Fig. 6). This spans from gene promoters that have high levels of unidirectional transcription and function mainly as orientation-dependent promoters (with little or no enhancer function) to elements with bidirectional (high level) transcription giving both promoter and enhancer orientation-independent activity (alternative promoters) to more distal elements with low levels of asymmetric or bidirectional transcription, which function mainly as enhancers, with a subset having weak orientation-independent promoter activity.

### How has dual enhancer and promoter activity evolved?

Bidirectionality has been suggested to be the ground state of transcription (Jin et al. 2017), and enhancer transcription may reflect this, serving as a source of evolutionary novelty. The finding that some TF-binding sites may possess the ability to initiate transcription (Nguyen et al. 2016) suggests that selection for enhancer activity could allow promoter activity to arise as a by-product. If the presence of low-level promoter activity either as a consequence of selection for enhancer activity or simply due to the relative nonspecificity of the transcriptional machinery is common in enhancer elements, then eRNA could be exploited by evolution for other purposes in transcriptional regulation, including coactivator activity (e.g., activation of CBP [Bose et al. 2017] or TF trapping at enhancers [Sigova et al. 2015]). Alternatively, transcribed enhancers may have evolved from promoters, where a promoter was duplicated and became separated from its target gene over evolutionary time. Gradually, the sequence features leading to strong promoter activity would become more degenerate, while the element may gain more TF-binding sites. Although there is currently no evidence of this, it would fit with the promoter and enhancer activity that we observed and with the fact that some species do not have distal enhancers but rather regulate gene expression by TF binding very close to the promoter. Although very speculative, alternative promoters may represent an intermediate state (from an evolutionary perspective) between promoters and enhancers. A previous study proposed that developmental enhancers evolve from inducible-type promoters (Arenas-Mena 2017). Of the elements that we tested, main gene promoters appear to have evolved to drive proximal orientation-dependent activation, possessing strong intrinsic promoter potential. At the other extreme, distal enhancers possess weak promoter potential but seem to have specialized toward a distal orientation-independent mode of action—a function achieved, presumably, through acquiring binding sites for a set of factors distinct from promoters. Distal enhancers themselves represent a heterogeneous population of elements with variable transcriptional properties. The coexistence of the two functions opens many questions: How can the same regulatory element facilitate enhancer and promoter function? Can one function be perturbed independently of the other? A preliminary answer to the latter is suggested here: Enhancer function was unaffected by changing orientation, while some promoter activity was lost, suggesting separate directional sequence determinants for these promoters’ activity.

## Materials and methods

Nuclei from unfixed frozen wild-type embryos (0.3–0.5 g) were extracted, and PRO-cap was performed according to Kwak et al. (2013) with minor modifications. To obtain mesoderm-specific CAGE libraries, mesodermal cells were FACS-purified from unfixed 6- to 8-h staged embryos expressing an EGFP under the control of an early mesodermal enhancer from the *twist* gene (Bonn et al. 2012b), and CAGE libraries were prepared as described in Schor et al. (2017). Biological replicates for both PRO-cap and CAGE came from independent embryo collections and RNA isolations. Samples were multiplexed and sequenced by an Illumina HiSeq 2000 sequencer (50-bp single-end reads). A detailed description of all of the data analysis is in the Supplemental Material.

For transgenic analysis, genomic regions tested in either the single readout vector or the dual enhancer–promoter assay are listed in Supplemental Table S9. All constructs were integrated at chromosomal position ZH-51C using the J27 landing site (Bischof et al. 2007). Homozygous embryos were formaldehyde-fixed and used for double fluorescent in situ hybridization using antisense probes against *lacZ* and *Mef2* (Fig. 3; Supplemental Fig. S6) or *lacZ* and *gfp* (Figs. 4, 5; Supplemental Fig. S7–S9). All images were taken with a Zeiss LSM 780 confocal microscope.

All raw sequencing data have been submitted to the European Molecular Biology Laboratory-European Bioinformatics Institute (EMBL-EBI) ArrayExpress database under accession numbers E-MTAB-6154 (PRO-cap; 3–4 and 6–8 h) and E-MTAB-6159 (meso-CAGE; 6–8 h). Processed files for visualization are available at http://furlonglab.embl.de/data.

## Supplementary Material

Supplemental Material
