# Supplementary material for: The degree of enhancer or promoter activity is reflected by the levels and directionality of eRNA transcription
Source: Genes Dev. 2018 Jan 1;32(1):42–57. doi: 10.1101/gad.308619.117 (PMC5828394; doi:10.1101/gad.308619.117)
Supplement: Supplemental Material [file supp_32_1_42__index.html]

Supplemental Material 

# The degree of enhancer or promoter activity is reflected by the levels and directionality of eRNA transcription

## Supplemental Material

- Supp\_Data\_File\_S1\_PROcap\_34hr.bw.zip
- Supp\_Data\_File\_S2\_PROcap\_68hr.bw.zip
- Supp\_Data\_File\_S3\_CAGE\_24hr.bw.zip
- Supp\_Data\_File\_S4\_CAGE\_68hr.bw.zip
- Supp\_Data\_File\_S5\_DHS\_eRNA.txt
- Supp\_Data\_File\_S6\_Enhancers\_eRNA.txt
- Supp\_Data\_File\_S7\_meso-CAGE\_68hr.bw.zip
- Supp\_Data\_File\_S8\_PROcap\_68hr\_enhancers\_CRMs.txt
- Supp\_Data\_File\_S9\_Primers\_Tested\_Regions.xlsx
- Supp\_Data\_File\_S10\_Transgenic\_Lines\_Summary.xlsx
- Supplemental\_Figures\_Methods.pdf
