# Supplementary material for: The degree of enhancer or promoter activity is reflected by the levels and directionality of eRNA transcription
Source: Genes Dev. 2018 Jan 1;32(1):42–57. doi: 10.1101/gad.308619.117 (PMC5828394; doi:10.1101/gad.308619.117)
Supplement: Supplemental Material [file supp_gad.308619.117_Supplemental_Figures_Methods.pdf]

SUPPLEMENTARY INFORMATION

The degree of enhancer or promoter activity is reflected by the levels and directionality of eRNA transcription

Olga Mikhaylichenko*, Vladyslav Bondarenko*, Dermot Harnett*, Ignacio E. Schor, Matilda Males, Rebecca R. Viales, Eileen E.M. Furlong†

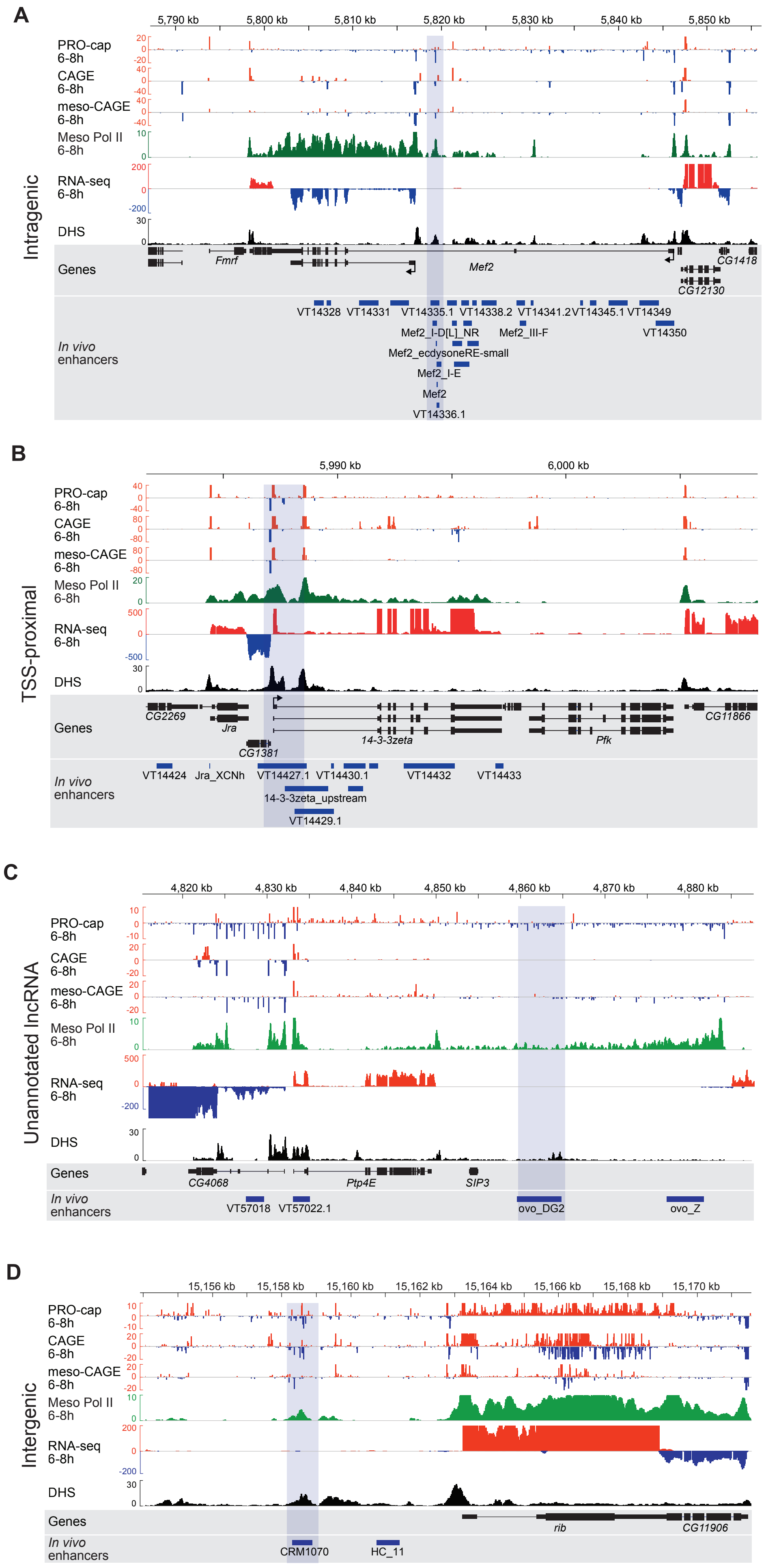
A
5,790 kb
5,800 kb
5,810 kb
5,820 kb
5,830 kb
5,840 kb
5,850 kb
Intragenic
PRO-cap 6-8h
CAGE 6-8h
meso-CAGE 6-8h
Meso Pol II 6-8h
RNA-seq 6-8h
DHS
Genes
Fmrf
Mef2
CG1418
CG12130
In vivo enhancers
VT14328 VT14331 VT14335.1 VT14338.2 VT14341.2 VT14345.1 VT14349
Mef2_I-D[L]_NR
Mef2_III-F
VT14350
Mef2_ecdysoneRE-small
Mef2_I-E
Mef2
VT14336.1
B
5,990 kb
6,000 kb
TSS-proximal
PRO-cap 6-8h
CAGE 6-8h
meso-CAGE 6-8h
Meso Pol II 6-8h
RNA-seq 6-8h
DHS
Genes
CG2269
Jra
CG1381
14-3-3zeta
Pfk
CG11866
In vivo enhancers
VT14424 Jra_XCNh VT14427.1 VT14430.1 VT14432 VT14433
14-3-3zeta_upstream
VT14429.1
C
4,820 kb
4,830 kb
4,840 kb
4,850 kb
4,860 kb
4,870 kb
4,880 kb
Unannotated lncRNA
PRO-cap 6-8h
CAGE 6-8h
meso-CAGE 6-8h
Meso Pol II 6-8h
RNA-seq 6-8h
DHS
Genes
CG4068
Ptp4E
SIP3
In vivo enhancers
VT57018 VT57022.1
ovo_DG2
ovo_Z
D
15,156 kb
15,158 kb
15,160 kb
15,162 kb
15,164 kb
15,166 kb
15,168 kb
15,170 kb
Intergenic
PRO-cap 6-8h
CAGE 6-8h
meso-CAGE 6-8h
Meso Pol II 6-8h
RNA-seq 6-8h
DHS
Genes
rib
CG11906
In vivo enhancers
CRM1070
HC_11

Figure S1. eRNA at enhancers located at different genomic positions

(A-D) Genome loci showing the variability of eRNA signal at enhancers located (A) within gene bodies (*VT14335* (Kvon et al. 2014)), (B) overlapping TSSs (*VT14427* (Kvon et al. 2014)), (C) within a putative unannotated lncRNA gene (*ovo_DG2* (Frankel et al. 2010)), and (D) distal to a gene (*CRM1070* (Zinzen et al. 2009)). Signal for mesoderm-specific Pol II occupancy at 6-8hr (Bonn et al. 2012a), RNA-seq 6-8hr (Brown et al. 2014), DHS, stage 11 (overlapping 6-8hr) (Thomas et al. 2010).

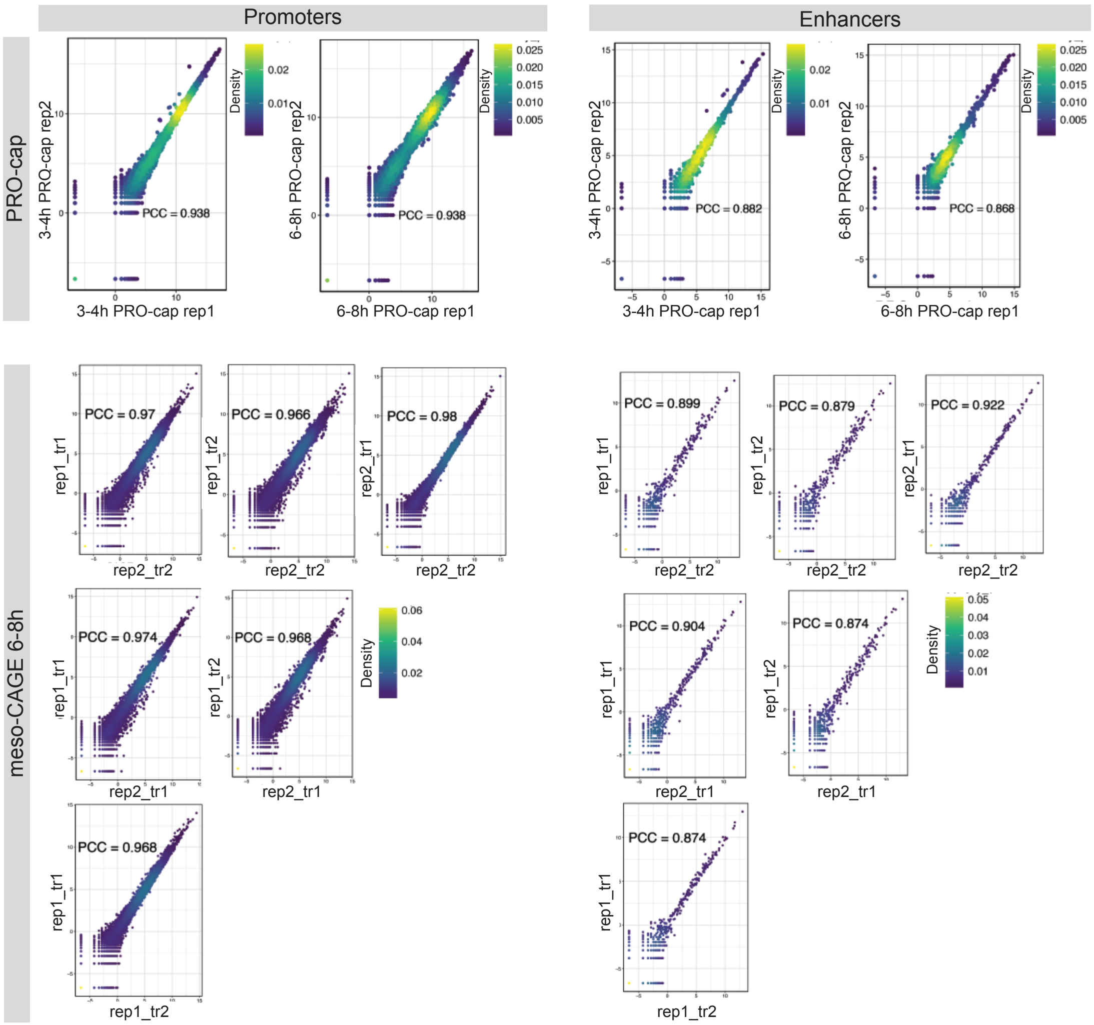

Figure S2. Quality of PRO-cap and Meso-CAGE data

Scatterplots (log2) of two independent biological replicates of PRO-cap (upper panels) and CAGE from FACS sorted mesodermal cells (lower panels), estimated at gene promoters (mRNA, ncRNA and pseudogenes), -200 bp to +300 bp around 5′ end of genes (n=16,157), and *in vivo* characterised intergenic enhancers (n = 1037, Table S6). For the meso-CAGE there are both biological and techniceal replicates (tr). Pearson correlation coefficents (PCC) are indicated.

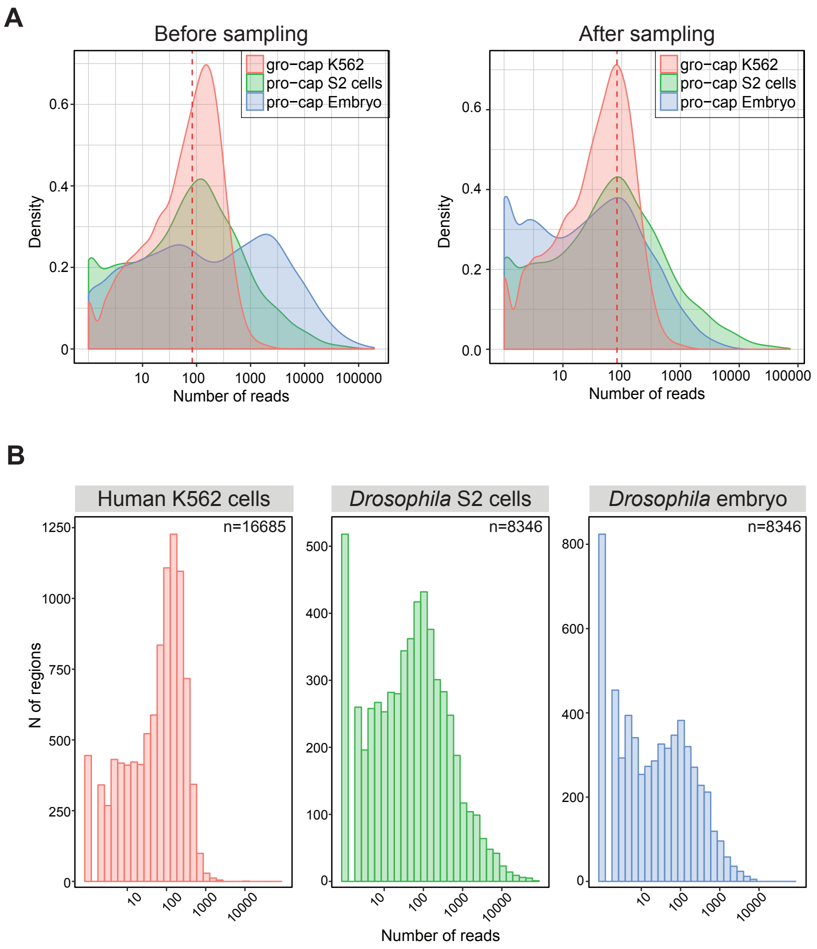

Figure S3. Subsampling human and *Drosophila* expression data

(A) Densities of read counts before subsampling (left) and after subsampling (right) in promoters, defined as 1kb regions around annotated 5′ gene ends (n = 16,685 and 8,346 for human and fly genomes, respectively) using human K562 cells GRO-cap (red, Core et al. 2014), *Drosophila* S2 cells PRO-cap (green, Core et al. 2014), and *Drosophila* embryo PRO-cap data (blue, 6-8hr, this study). Median value in (A) is depicted with a dashed red line.   **(B)** Read densities of GRO-cap (human K562 cells (Core et al. 2014)) and PRO-cap (*Drosophila* S2 cells and embryos (6-8hr)) in promoters, defined as 1kb regions around annotated 5′ gene ends (n = 16,685 and 8,346 for human and fly genomes, respectively).

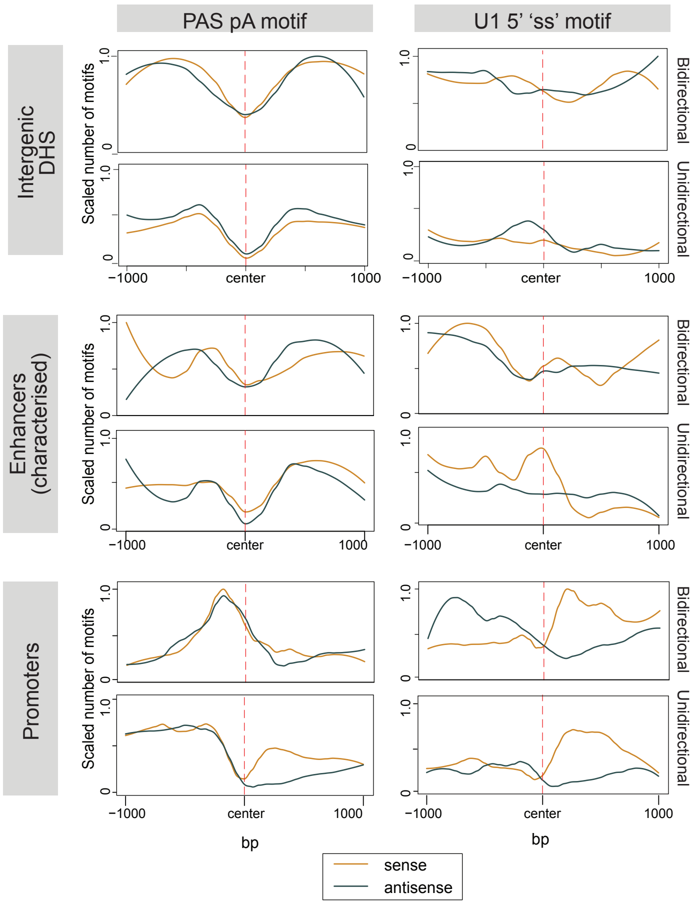

Figure S4. Enrichment of U1/PAS motifs at *Drosophila* promoters and enhancers

U1 splice site PWM was constructed using 11 bases (on the sense strand) at the 5′-ends of all introns (4 bases of a neighbouring exon and 7 bases of an intron). To define polyadenylation signals (PAS), we generated all possible k-mers of the PAS AATAAA motif, with maximum 2 mismatches, and considered their distribution up to 100 bases upstream of 3′ ends of all transcripts, using *Drosophila* annotation v5.57. PAS sites (such as AATAAA) have a maximum density 20-25 bases downstream of the annotated Transcriptional End Site (TES, Almada et al. 2013). Distributions of all k-mers near 3′ ends were compared with the distribution of AATAAA motif using Pearson correlation coefficient (PCC). k-mers with PCC >0.7 were ranked based on their PCC values and used to count pA site usage. Genome-wide motifs search for U1 and pA (AATAAA/ATTAAA) PWMs was performed with FIMO from the MEME-Suite (Grant et al. 2011). For U1, PWMs with a p-value < 0.001 was used (matches with a score lower 4 were discarded). For PAS, all exact matches to either AATAAA or ATTAAA DNA sequences were used, corresponding to ~75% of 6-mers used as cleavage sites in mammalian genomes (Almada et al. 2013), which is comparable to our estimate for *Drosophila*. Within each panel, top and bottom plots correspond to bi- (OI< 0.6) and unidirectionally (OI> 0.8) transcribed elements, respectively.

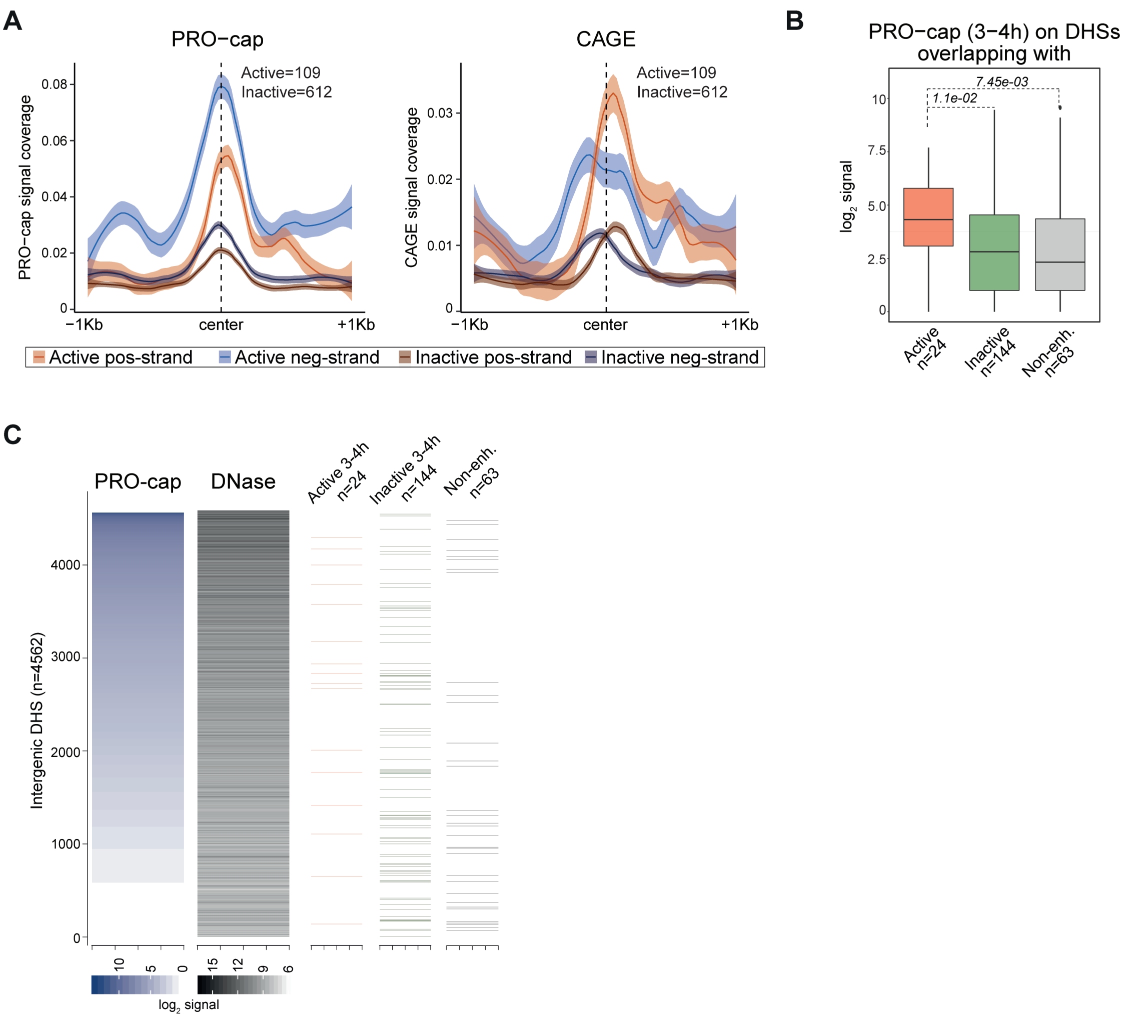

Figure S5. eRNA transcription is correlated with enhancer activity at early stages of development (3-4hr)

(A-C) Levels of eRNA transcription centered on DHS within characterised developmental enhancers (both inter- and intragenic) in an active or inactive state. **(A, B)** Embryonic PRO-cap (3-4hr) and CAGE (2-4hr, (Schor et al. 2017)) signal at enhancers that are active at 3-4hr in any embryonic tissue or inactive at 3-4hr, but active at other time points. **(C)** Boxplots show levels of eRNA transcripiton (log2, PRO-cap) at 3-4hr in intergenic DHS within active characterised enhancers (red), inactive enhancers at 3-4hr (green), and non-enhancer regions (grey). *P*-values from two-sided Wilcoxon test. **(D)** Heatmap showing ranked eRNA signal (PRO-cap 3-4hr) and DHS (stage 5, overlapping 2-4hr) signal (log2 of the sum of reads per region) over all intergenic DHS (n=4,562, Table S3). The position of intergenic enhancers active or inactive at 6-8hr, and non-enhancers are shown.

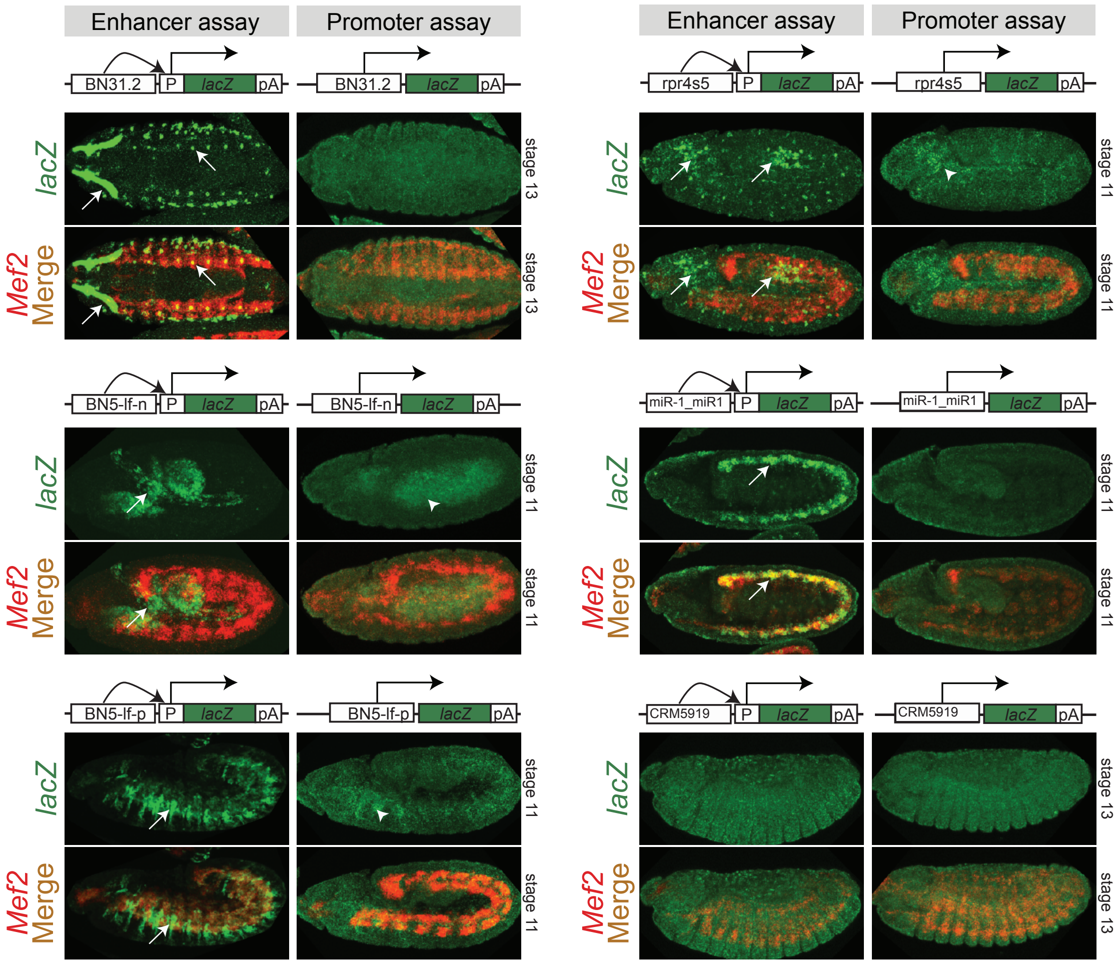

Figure S6. Transcribing enhancers can act as promoters *in vivo*

Double *in situ* hybridization against the *lacZ* reporter gene, driven by the tested element (green) in the presence or absence of a minimal promoter (*hsp70)*, and a mesoderm marker gene, *Mef2* (red). *Left panel*: elements with "stable" endogenous eRNA (*BN31.2, BN5-lf-p, BN5-lf-n* (Zinzen et al. 2009)). *Right panel*: elements with "unstable" endogenous eRNA (*rpr4s5* (Lohmann 2003)*, miR-1_miR-1* (Biemar et al. 2005)*, CRM5919*). Embryos are laterally (dorsally – BN31.2) oriented with anterior to the left. *BN5-lf-p, BN5-lf-n* and *rpr4s5* produced weak levels of transcript in the absence of a minimal promoter (white arrow head). *BN31.2, CRM669 and miR-1_miR-1* had no detectable promoter activity. Enhancer activity is indicated by the white arrows.

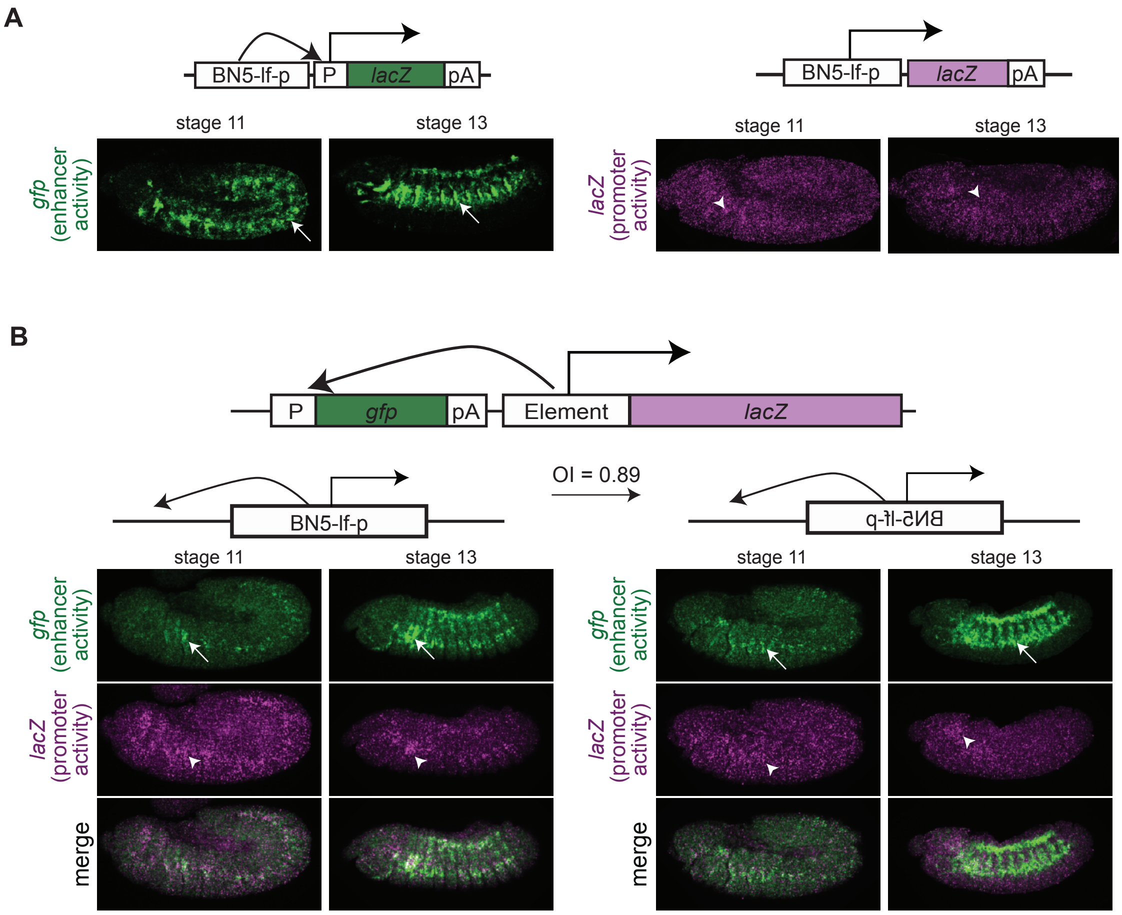

Figure S7. **Enhancer-gene distance affects the levels of reporter expression but not its spatio-temporal pattern**

(A) Single assays, measuring enhancer and promoter activity separately, placing the enhancer directly upstream from the reporter, as typically done. *In situ* hybridization against the *lacZ* reporter gene driven by the *BN5-lf-p* element (green). *BN5-lf-p* acts as an enhancer (green), driving expression in the somatic muscle from stage 10, as well as in the lateral ectoderm (white arrow). It has weak promoter activity (magenta) restricted to the same pattern (white arrowhead). **(B)** Dual enhancer-promoter activity vector: Double *in situ* hybridization against *gfp* driven by the *hsp70* minimal promoter under the control of *BN5-lf-p* (green, enhancer assay) and *lacZ* driven by *BN5-lf-p* in the absence of a minimal promoter (magenta, promoter assay). *BN5-lf-p* placed in the dual E-P vector produces somatic muscle expression (white arrow) in an orientation-independent manner, as well as weak promoter activity in both orientations (white arrowhead). Stronger promoter expression is detected in the "plus" (sense) orientation (*in-situ* and imaging conditons are identical between sense and antisense). OI=Orientation Index, with 0.89 being largely unidirectional transcription.

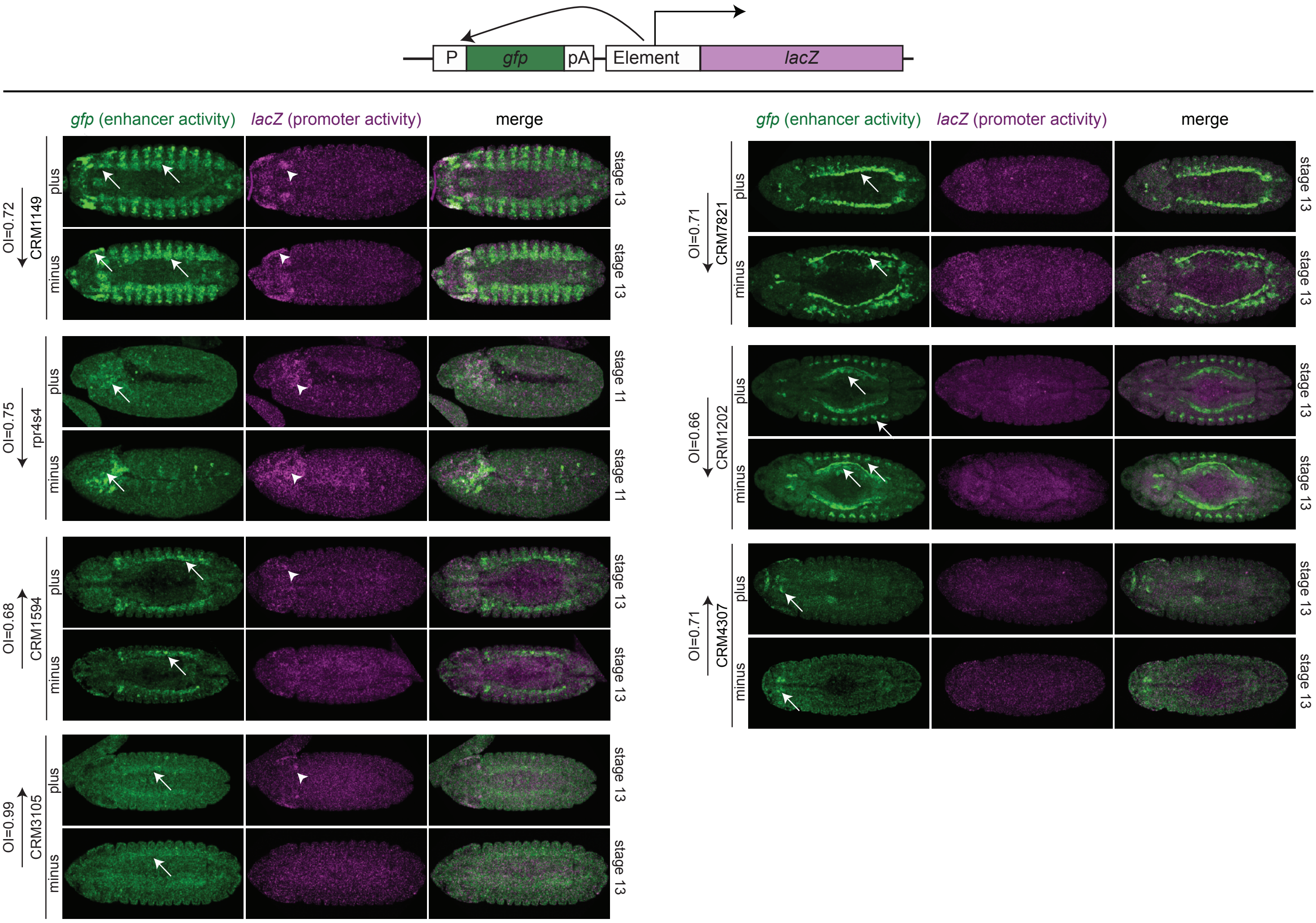

Figure S8. Intergenic enhancers generally act as promoters in both orientations

Double *in situ* hybridization against the *gfp* reporter gene driven by a minimal promoter under the control of the tested elements (green, enhancer assay) and *lacZ* driven by the tested elements without a minimal promoter (magenta, promoter assay). Each element was assayed in both orientations; "plus" and "minus" denote the DNA strand. All elments function as enhancers (green), driving expression in different tissues (white arrows). *Left panel*: *CRM1149* and *rpr4s4* act as promoters, driving *lacZ* expression in both orientations, in part of the brain (CRM1149) and head (rpr4s4), white arrows, which overlaps part of the enhancers activity. *CRM1594* and *CRM3105* drive weak lacZ expression (promoter activity) in the brain (white arrowheads), in only one orientation. *Right panel*: *CRM7821, CRM4307*, *CRM1202* have no detectable promoter activity, while acting as enhancers in the somatic muscle, visceral muscle, and anterior foregut, respectively (white arrows). Embryos are laterally (stage 11) or dorsally (stage 13) oriented with anterior to the left. OI=Orientation Index value, black arrow denotes the main orientation of endogenous transcription: upwards arrow = "plus" DNA strand, downwards arrow ="minus" DNA strand.

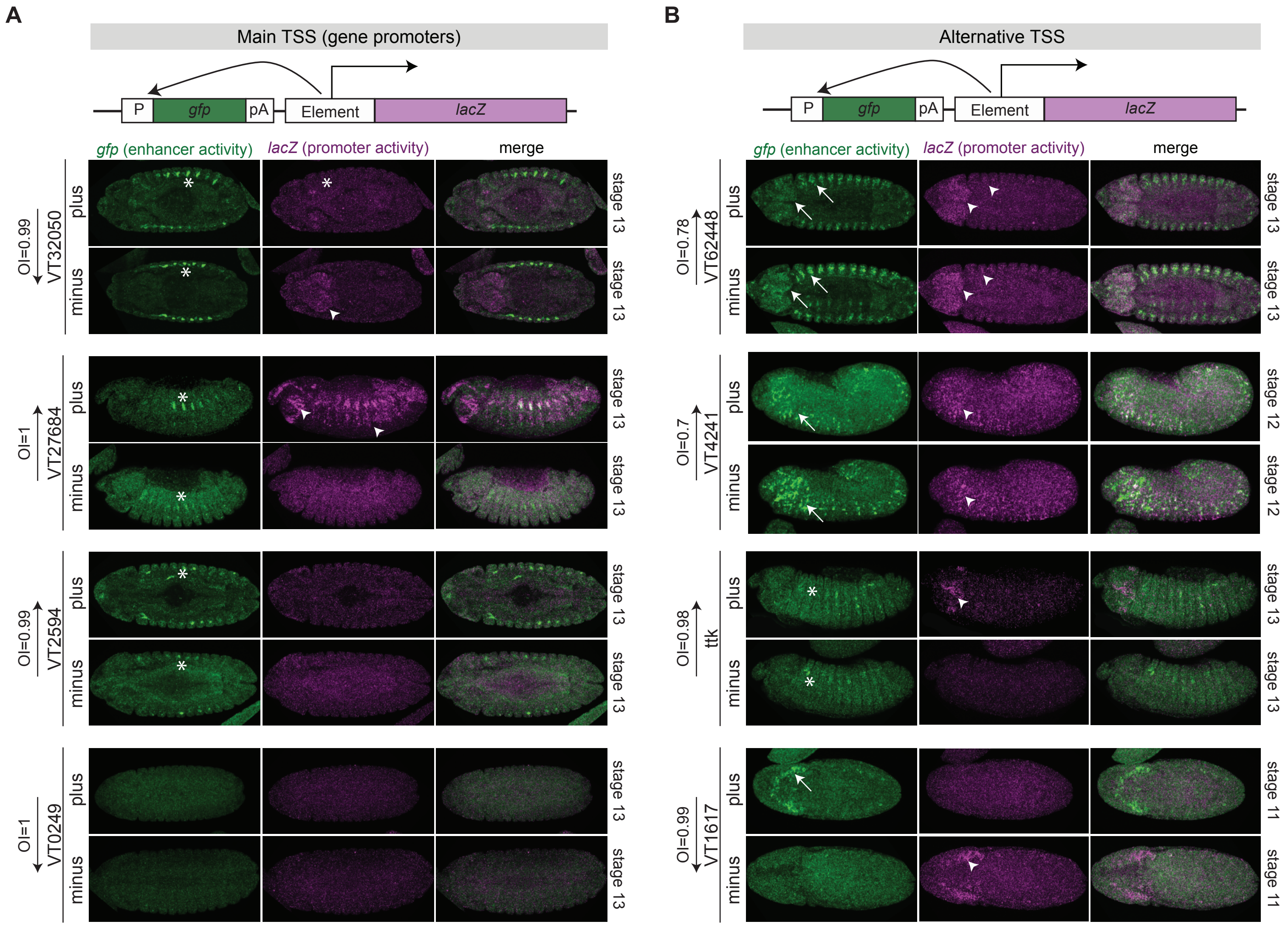

Figure S9. Alternative promoters function as developmental enhancers *in vivo*

(A, B) Double *in situ* hybridization against the *gfp* reporter gene driven by a minimal promoter under the control of the tested element (green, enhancer assay) and *lacZ* driven by the tested element without a minimal promoter (magenta, promoter assay). **(A)** The four main promoters do not function as enhancers. Three promoter elements, overlapping main TSSs (*VT32050*, *VT27684*, *VT2594*) produced similar *gfp* expression patterns in segmental ectodermal strips characteristic for the J27 line, and were therefore considered as background activity (asterisk). *VT0249* did not produce any expression. *VT32050* and *VT27684* act as promoters in the central nervous system and visceral/somatic muscle, respectively (white arrowheads). **(B)** Three elements overlapping alternative TSSs (*VT62448, VT4241, VT1617*) have both enhancer (white arrows) and promoter (white arrow heads) activities. The *ttk* element (overlapping a unidirectional alternative TSS), only acts as a weak promoter in an orientation-dependent manner. Embryos are laterally (stages 11 or 12) or ventrally (stage 13) oriented with anterior to the left. OI=Orientation Index value, black arrow denotes the main orientation of endogenous transcription: upwards arrow = “plus” DNA strand, downwards arrow =“minus” DNA strand.

SUPPLEMENTARY METHODS

Processing and classification of DNase l hypersensitivity sites (DHS)

Using stage-specific whole embryo (WE) DNase-seq data from (Thomas et al., 2010) we recalled peaks using hotspot (John et al., 2011) with an FDR of 0.05. Peak summits (150bp long) that intersect between biological replicates from the same developmental stage were merged and used for downstream analysis as DNase l hypersensitivity sites (DHS). DHS were classified as promoter regions (TSS-proximal), intragenic and intergenic as follows: To define promoter regions we used unambiguous TSS-to-gene assignments (n = 24,264) from Batut *et al* based on RAMPAGE (Batut, Dobin, Plessy, Carninci, & Gingeras, 2012). Genomic locations of TSS (RAMPAGE peaks, (Batut et al., 2012)) were extracted from FlyBase (ID = FBlc0000537). In addition to annotated TSS, this dataset allowed us to account for alternative transcription initiation sites, which are commonly used during development, but may not be present in the current genome annotation (Batut et al., 2012). For each gene, the RAMPAGE TSS peak with maximum CAGE signal (using our 6-8hr data), across all assigned TSS for that gene (Batut et al., 2012). was classified as 'main', while the remaining TSS were classified as 'alternative'. For intragenic elements, we required that a non TSS-proximal DHS is located entirely within a gene from FlyBase annotation (v5.57) or lncRNA (Young et al., 2012). Intergenic elements were defined as being located > 500bp and 1.5 kb away from the 5′ and 3′ ends of genes, respectively (FlyBase annotation v5.57 and lncRNAs (Young et al., 2012)). To obtain a unique master set of intergenic non-TSS DHS, we merged DHS from all stages (5, 9, 11,13), resulting in 4,562 non-TSS intergenic DHS regions. All DHS regions, with their classification (promoter, intra- and inter-genic) and RNA signal (PRO-cap, CAGE, meso-CAGE), used for the analysis in Fig. 2D, E and Fig. S5B, C, are provided in Table S5.

Compiling a compendium of uniformly sized characterised developmental enhancers

We compiled a collection of previously characterised enhancers from Kvon *et al* (mainly neuronal) (Kvon et al., 2014), REDfly (Gallo et al., 2011) and from our own lab (mainly mesodermal). Embryonic stages were matched to time points as follows: stage 4-6 to 3-4hr, stage 7-8 to 4-6hr, stage 9-10 and 11-12 to 6-8hr, stage 13-16 to 10-12hr (Zinzen, Girardot, Gagneur, Braun, & Furlong, 2009). As these enhancers were collated from different sources, they have very different lengths that do not necessarily represent the 'centre' (or 'peak') of biological activity such as the site of transcription factors (TF) binding. For example, all the

Vienna Tiles are approximately 2kb, enhancers from REDfly can range from a few hundred base pairs to over 3kb and the Furlong lab mesodermal enhancers are 400bp on average. We therefore resized each enhancer region, based on its DHS peak (using the DNase-seq data described above), requiring that >90% of the DHS length is contained within it. All enhancer regions with more than one DHS were excluded, as they likely represent two enhancer elements, which might differ in their spatio-temporal activity. This resulted in a stringent set of 1,055 single DHS enhancers with *in vivo* characterised activity. The point of maximum DHS signal (summing DNase-seq coverage from all stages) was used as the enhancer centre. 500bp up- and downstream relative to this central point were used to define a uniformly sized (1kb) set of developmental enhancers whose spatio-temporal activity has been assessed at all stages of embryogenesis. From this set, we excluded 18 enhancers that overlapped by >50% of their length to avoid bias due to double counting of reads. The remaining 1,037 enhancers (Table S6) were used to select characterised enhancers that are active or inactive at different developmental stages (Fig. 2A, B, Fig S5A) or tissues (Fig. 2C). As described above, these enhancers were classified as intergenic (n=220), intragenic (n=497), and TSS-proximal (n=198). 122 enhancers were not assigned to any classes according to our definitions.

PRO-cap and meso-CAGE library preparation

Nuclei from unfixed, frozen wild type embryos (0.3-0.5g) were extracted according to Saunders *et al.* (Saunders, Core, Sutcliffe, Lis, & Ashe, 2013). PRO-cap was performed according to Kwak *et al.* (Kwak, Fuda, Core, & Lis, 2013), with minor modifications. Libraries were amplified using 16 PCR cycles, and the CAGE purification step was replaced by size selection with AMPure bead purification. To obtain mesoderm-specific CAGE libraries, mesodermal cells were FACS-purified from unfixed, 6-8hr staged embryos expressing an EGFP-tagged CBP20 protein under the control of an early mesodermal enhancer from the *twist* gene (Bonn et al., 2012). RNA was isolated and CAGE libraries prepared from 2.5 ug of total RNA as described in Schor *et al.* (Schor et al., 2017). Biological replicates for both PRO-cap and CAGE came from independent embryo collections and RNA isolations, while technical replicates came from independent CAGE libraries made from the same RNA. Samples were multiplexed and sequenced by an Illumina HiSeq 2000 sequencer (50bp single-end reads).

Processing PRO-cap and CAGE data

Illumina sequence files were demultiplexed and converted to FASTQ. Sequencing quality was assessed using FastQC as provided in Galaxy (Blankenberg et al., 2010; Giardine et al., 2005; Goecks, Nekrutenko, Taylor, Galaxy Team, 2010), tool version 1.0.0. Reads were aligned against the *D. melanogaster* dm3 genome using bwa mem (Li et al., 2009) (version 0.7.12) with default parameters. Given the high reproducibility between replicates (Fig. S2), technical and biological replicates were merged resulting in 66,085,730 and 52,422,944 reads for 3-4hr and 6-8hr time points, respectively and 109,285,610 reads for mesodermal CAGE data. The whole embryo CAGE data processing is described in Schor *et al* (Schor et al., 2017). For this analysis, we combined CAGE reads from all samples at equivalent time-points, which resulted in 1,045 million mapped reads. For data visualization, we extracted positions of 5′ ends of reads and converted them into BigWig file format with a custom R script (provided in Tables S1-4 and S7). The provided Bigwig files represent counts of these 5′ sites per base pair. Signal columns in tables (Tables S5, S6, S8) are derived from counts of read 5′ ends.

Comparing eRNA levels and directionality between *Drosophila* and human

To compare the relative levels of eRNA on regulatory elements between species, we first made the data comparable by ensuring that the level of RNA signal was similar on promoters, taking care of different sequencing depths between samples. We randomly sampled GRO-cap (K562) (Core et al., 2014), PRO-cap (S2 cells, GSM1032759) (Kwak et al., 2013), and our PRO-cap (whole embryo) data so that the read counts per promoter regions align between all datasets (Fig. S3). For both human and fly, only protein-coding genes were used: 5′ gene ends were extended to +/-0.5 kb regions, excluding intersections with other TSSs on the opposite strand and RAMPAGE peaks (N=16,685 for human and 8,346 for fly). Using the resampled data, we then compared read counts over DHS defined identically for both *Drosophila* S2 and human K562 cell lines, taking the DHS data from (Arnold et al., 2013) and ENCODE (ENCODE Project Consortium, 2012), respectively. Only intergenic regions (resized to 1kb prior to analysis), located at least 0.5 kb and 1.5 kb away of gene 5′ and 3′ ends, respectively, were used for the analysis (Fig. 1A, Fig. S3).

To compare directionality of eRNA transcripts, we calculated the orientation index (OI) (Core et al., 2012) as the fraction of total reads over a region found in the sense orientation. Here, sense orientation for both promoters and enhancers was defined as the one with greatest read count (Fig. 1B).

Motifs enrichment analysis

Inr motif analysis was performed by centering on the maximal point of transcription. First, we divided intergenic DHS (putative enhancers) into unidirectional and bidirectional based on their OI values (OI > 0.8 and <0.6, respectively) and performed a PWM search using FIMO (Grant, Bailey, & Noble, 2011), with a p-value threshold <1E-4. Centrimo (Bailey & Machanick, 2012) with minimum acceptable match score 5.0 and e-value ≤ 10 was used assess the positional enrichment of the INR motif (Fig. 1C).

eRNA levels on characterised enhancers in an active and inactive state

For PRO-cap and CAGE data, the summed signal was estimated over 20 bp bins resulting in 100 points per enhancer (from -1kb to +1kb of the enhancer centre). Points with the top and bottom 5% of signal intensity (estimated from all binned regions) were excluded to avoid the impact of outliers. Due to high resolution of the PRO-cap and CAGE data, 95% confidence intervals of the trimmed mean were calculated, assuming a normal distribution, followed by Lowess smoothing of the mean values. Scaled values (used in Fig. 2A-C, Fig S5A) were obtained by scaling the minimum average intensity value and then dividing by the maximum average intensity for each separate data type, over the bins within the region from +/-1kb of the enhancer centre.

Transgenic reporter assays

To assay for enhancer and promoter activity using the single read-out vector (Fig. 3, Fig. S6), corresponding genomic regions were amplified by PCR (primers listed in Table S9) and placed either upstream of a minimal *hsp70* promoter driving a *lacZ* reporter gene (enhancer assay) or directly upstream of the *lacZ* reporter gene with no promoter (promoter assay) in a modified pH Pelican vector (Barolo, Carver, & Posakony, 2000). For the dual assay, genomic regions were amplified by PCR (Table S9), digested with Hind III restriction enzyme and ligated into the multiple cloning site of the dual assay vector. Derived bacterial colonies were screened by colony PCR to obtain clones with inserts in both orientations.

All constructs were injected according to standard methods into the landing site line J27 (Bischof, Maeda, Hediger, Karch, & Basler, 2007), yielding integration at chromosomal position ZH-51C. Embryos from homozygous lines were formaldehyde-fixed and stained by

double fluorescent *in situ* hybridization (Furlong, Andersen, Null, White, & Scott, 2001) using anti-sense probes against *lacZ* and *Mef2* (Fig. 3, Fig. S6) or *lacZ* and *gfp* (Fig. 4,5, S7-9). All images were taken with a Zeiss LSM 780 confocal microscope.

NGS Data availability

Raw sequence data has been deposited in the EMBL-EBI ArrayExpress database under accession numbers E-MTAB-6154 (PRO-cap, 3-4hr and 6-8hr) and E-MTAB-6159 (meso-CAGE 6-8hr). Processed files for visualization are available at: http://furlonglab.embl.de/data/

References

Arnold, C. D., Gerlach, D., Stelzer, C., Boryn, L. M., Rath, M., & Stark, A. (2013). Genome-Wide Quantitative Enhancer Activity Maps Identified by STARR-seq. *Science*, *339*(6123), 1074–1077. http://doi.org/10.1126/science.1232542

Bailey, T. L., & Machanick, P. (2012). Inferring direct DNA binding from ChIP-seq. *Nucleic Acids Research*, *40*(17), e128–e128. http://doi.org/10.1093/nar/gks433

Barolo, S., Carver, L. A., & Posakony, J. W. (2000). GFP and beta-galactosidase transformation vectors for promoter/enhancer analysis in Drosophila. *BioTechniques*, *29*(4), 726–728– 730– 732.

Batut, P., Dobin, A., Plessy, C., Carninci, P., & Gingeras, T. R. (2012). High-fidelity promoter profiling reveals widespread alternative promoter usage and transposon-driven developmental gene expression. *Genome Research*, *23*(1), 169–180. http://doi.org/10.1101/gr.139618.112

Bischof, J., Maeda, R. K., Hediger, M., Karch, F., & Basler, K. (2007). An optimized transgenesis system for Drosophila using germ-line-specific phiC31 integrases. *Pnas*, *104*(9), 3312–3317. http://doi.org/10.1073/pnas.0611511104

Blankenberg, D., Kuster, Von, G., Coraor, N., Ananda, G., Lazarus, R., Mangan, M., et al. (2010). Galaxy: a web-based genome analysis tool for experimentalists. *Current Protocols in Molecular Biology / Edited by Frederick M. Ausubel ... [Et Al.]*, *Chapter 19*, Unit 19.10.1–21. http://doi.org/10.1002/0471142727.mb1910s89

Bonn, S., Zinzen, R. P., Perez-Gonzalez, A., Riddell, A., Gavin, A.-C., & Furlong, E. E. M. (2012). Cell type-specific chromatin immunoprecipitation from multicellular complex samples using BiTS-ChIP. *Nature Protocols*, *7*(5), 978–994. http://doi.org/10.1038/nprot.2012.049

Core, L. J., Martins, A. L., Danko, C. G., Waters, C. T., Siepel, A., & Lis, J. T. (2014). Analysis of nascent RNA identifies a unified architecture of initiation regions at mammalian promoters and enhancers. *Nature Genetics*, *46*(12), 1311–1320. http://doi.org/10.1038/ng.3142

Core, L. J., Waterfall, J. J., Gilchrist, D. A., Fargo, D. C., Kwak, H., Adelman, K., & Lis, J. T. (2012). Defining the status of RNA polymerase at promoters. *CellReports*, *2*(4), 1025–

1035. http://doi.org/10.1016/j.celrep.2012.08.034

ENCODE Project Consortium. (2012). An integrated encyclopedia of DNA elements in the human genome. *Nature*, *489*(7414), 57–74. http://doi.org/10.1038/nature11247

Furlong, E. E., Andersen, E. C., Null, B., White, K. P., & Scott, M. P. (2001). Patterns of gene expression during Drosophila mesoderm development. *Science*, *293*(5535), 1629–1633. http://doi.org/10.1126/science.1062660

Gallo, S. M., Gerrard, D. T., Miner, D., Simich, M., Soye, Des, B., Bergman, C. M., & Halfon, M. S. (2011). REDfly v3.0: toward a comprehensive database of transcriptional regulatory elements in Drosophila. *Nucleic Acids Research*, *39*(Database issue), D118–23. http://doi.org/10.1093/nar/gkq999

Giardine, B., Riemer, C., Hardison, R. C., Burhans, R., Elnitski, L., Shah, P., et al. (2005). Galaxy: a platform for interactive large-scale genome analysis. *Genome Research*, *15*(10), 1451–1455. http://doi.org/10.1101/gr.4086505

Goecks, J., Nekrutenko, A., Taylor, J., Galaxy Team. (2010). Galaxy: a comprehensive approach for supporting accessible, reproducible, and transparent computational research in the life sciences. *Genome Biology (Online Edition)*, *11*(8), R86. http://doi.org/10.1186/gb-2010-11-8-r86

Grant, C. E., Bailey, T. L., & Noble, W. S. (2011). FIMO: scanning for occurrences of a given motif. *Bioinformatics (Oxford, England)*, *27*(7), 1017–1018. http://doi.org/10.1093/bioinformatics/btr064

John, S., Sabo, P. J., Thurman, R. E., Sung, M.-H., Biddie, S. C., Johnson, T. A., et al. (2011). Chromatin accessibility pre-determines glucocorticoid receptor binding patterns. *Nature Genetics*, *43*(3), 264–268. http://doi.org/10.1038/ng.759

Kvon, E. Z., Kazmar, T., Stampfel, G., Yáñez-Cuna, J. O., Pagani, M., Schernhuber, K., et al. (2014). Genome-scale functional characterization of Drosophila developmental enhancers in vivo. *Nature*. http://doi.org/10.1038/nature13395

Kwak, H., Fuda, N. J., Core, L. J., & Lis, J. T. (2013). Precise Maps of RNA Polymerase Reveal How Promoters Direct Initiation and Pausing. *Science*, *339*(6122), 950–953. http://doi.org/10.1126/science.1229386

Li, H., Handsaker, B., Wysoker, A., Fennell, T., Ruan, J., Homer, N., et al. (2009). The Sequence Alignment/Map format and SAMtools. *Bioinformatics (Oxford, England)*, *25*(16), 2078–2079. http://doi.org/10.1093/bioinformatics/btp352

Saunders, A., Core, L. J., Sutcliffe, C., Lis, J. T., & Ashe, H. L. (2013). Extensive polymerase pausing during Drosophila axis patterning enables high-level and pliable transcription. *Genes & Development*, *27*(10), 1146–1158. http://doi.org/10.1101/gad.215459.113

Schor, I. E., Degner, J. F., Harnett, D., Cannavò, E., Casale, F. P., Shim, H., et al. (2017). Promoter shape varies across populations and affects promoter evolution and expression noise. *Nature Genetics*, *49*(4), 550–558. http://doi.org/10.1038/ng.3791

Thomas, S., Li, X.-Y., Sabo, P. J., Sandstrom, R., Thurman, R. E., Canfield, T. K., et al. (2010). Dynamic reprogramming of chromatin accessibility during Drosophila embryo development. *Genome Biology (Online Edition)*, *12*(5), R43–R43. http://doi.org/10.1186/gb-2011-12-5-r43

Young, R. S., Marques, A. C., Tibbit, C., Haerty, W., Bassett, A. R., Liu, J.-L., & Ponting, C.

P. (2012). Identification and properties of 1,119 candidate lincRNA loci in the Drosophila melanogaster genome. *Genome Biology and Evolution*, *4*(4), 427–442. http://doi.org/10.1093/gbe/evs020

Zinzen, R. P., Girardot, C., Gagneur, J., Braun, M., & Furlong, E. E. M. (2009). Combinatorial binding predicts spatio-temporal cis-regulatory activity. *Nature*, *462*(7269), 65–70. http://doi.org/10.1038/nature08531
